# Impact of nuclear Piwi elimination on chromatin state in *Drosophila melanogaster* ovaries

**DOI:** 10.1093/nar/gku268

**Published:** 2014-04-29

**Authors:** Mikhail S. Klenov, Sergey A. Lavrov, Alina P. Korbut, Anastasia D. Stolyarenko, Evgeny Y. Yakushev, Michael Reuter, Ramesh S. Pillai, Vladimir A. Gvozdev

**Affiliations:** 1 Institute of Molecular Genetics, Russian Academy of Sciences, Moscow 123182, Russia; 2 European Molecular Biology Laboratory, Grenoble Outstation, 71 avenue des Martyrs, 38042 France; 3 Unit for Virus Host-Cell Interactions, University of Grenoble Alpes-EMBL-CNRS, 71 avenue des Martyrs, 38042 France

## Abstract

The Piwi-interacting RNA (piRNA)-interacting Piwi protein is involved in transcriptional silencing of transposable elements in ovaries of *Drosophila melanogaster*. Here we characterized the genome-wide effect of nuclear Piwi elimination on the presence of the heterochromatic H3K9me3 mark and HP1a, as well as on the transcription-associated mark H3K4me2. Our results demonstrate that a significant increase in the H3K4me2 level upon nuclear Piwi loss is not accompanied by the alterations in H3K9me3 and HP1a levels for several germline-expressed transposons, suggesting that in this case Piwi prevents transcription by a mechanism distinct from H3K9 methylation. We found that the targets of Piwi-dependent chromatin repression are mainly related to the elements that display a higher level of H3K4me2 modification in the absence of silencing, i.e. most actively transcribed elements. We also show that Piwi-guided silencing does not significantly influence the chromatin state of dual-strand piRNA-producing clusters. In addition, host protein-coding gene expression is essentially not affected due to the nuclear Piwi elimination, but we noted an increase in small nuclear spliceosomal RNAs abundance and propose Piwi involvement in their post-transcriptional regulation. Our work reveals new aspects of transposon silencing in *Drosophila*, indicating that transcription of transposons can underpin their Piwi dependent silencing, while canonical heterochromatin marks are not obligatory for their repression.

## INTRODUCTION

Argonaute-associated small RNAs cause post-transcriptional gene silencing or regulate target genes at the level of transcription. Transcriptional gene silencing (TGS) by small RNAs has been shown in different organisms, including plants, fungi and animals (1,2). The most deeply studied system of small RNA-guided TGS is represented by heterochromatic repeats in fission yeast Schizosaccharomyces pombe, where the AGO1 protein forms the RNA-induced transcriptional silencing complex that interacts with nascent target RNAs and recruits the H3K9 histone methyltransferase complex. H3K9 methylation leads to the binding of an HP1 protein homolog (Swi6) and triggers heterochromatin formation (1,3). A special class of small RNAs called Piwi-interacting RNAs (piRNAs) is directed mainly to the repression of transposable elements in animal gonads (1,2,4). In vertebrates piRNAs induce TGS coupled with DNA methylation (5,6). The role of the piRNA pathway in TGS of transposons has also been demonstrated in ovaries of *Drosophila melanogaster* lacking noticeable DNA methylation (7–14). Three piRNA-binding proteins of the PIWI subfamily are involved in transposon suppression in ovaries of *D. melanogaster*: Piwi located in the nuclei in both germinal and somatic ovarian cells, and Aub and Ago3 functioning in the cytoplasm of germinal cells (15,16). Early data have revealed the role of Piwi in transcriptional silencing of transgenes in *D. melanogaster* somatic tissues (17,18). Then the Piwi protein was shown to repress several transposons in ovaries via establishment of the chromatin state, which is associated with the recruitment of the known heterochromatic marks H3K9me2/3 and HP1a (9,10), and HP1a knockdown was shown to upregulate transposons in the germline (9). Recently, several groups demonstrated the role of Piwi in transcriptional silencing of transposons by genome-wide deep sequencing approaches (11–14). In particular, a pronounced increase of Pol II occupancy and a decrease of H3K9me3 mark in transposon bodies was shown upon Piwi depletion. At least two nuclear proteins (Maelstrom and Gtsf1) have been identified as putative Piwi cofactors in transcriptional silencing (11,19–21). It is thought that Piwi binds to nascent RNA via its piRNA guide and recruits H3K9 histone methyltransferase to target genomic loci, similarly to AGO1-mediated repression in S. pombe (22). It is generally accepted that H3K9 methylation results in HP1 binding that leads to the establishment of heterochromatic state, preventing transcription (23). However, the studies of transcriptional silencing in somatic gonadal cells revealed that the presence of H3K9me3 signal allows the passage of transposon transcription, suggesting it is not the final silencing mark (11).

The data concerning the specificity of Piwi-mediated TGS, in particular its influence on genome targets beyond transposons, are contradictory (11,13,14). One report based mainly on the analysis of piwi null mutants showed that Piwi may act as a global regulator of genome transcription in ovaries and most transposons are targets of Piwi transcriptional silencing (14). The use of Piwi RNAi knockdowns in germinal ovarian cells demonstrated that only a limited number of transposon families are regulated by Piwi (12,13). However, it has been emphasized that the RNAi response had not started yet in early-stage egg chambers (12). Thus, some Piwi-dependent chromatin modifications can be continuously inherited epigenetically and this approach to reveal Piwi effects may distort its true impact on chromatin status. On the other hand, an adequate analysis of piwi null mutants is significantly complicated by their phenotype, since the ovarioles in these mutants contain very few or no egg chambers due to Piwi influence on germline stem cell maintenance and differentiation (24,25). Here, we investigated the effect of the absence of nuclear Piwi on the chromatin of ovaries due to the piwi^Nt^ mutation, removing the Piwi NLS signal (Nuclear Localization Signal). piwi^Nt^ flies completely lose the silencing function of the Piwi protein, but retain the processes of germline stem cell self-renewal and differentiation, showing near-normal morphology of ovaries (10). The effect of this mutation on transposon chromatin in ovaries has been previously analyzed by chromatin immunoprecipitation-polymerase chain reaction (ChIP-PCR) and interpreted in favor of nuclear/transcriptional silencing mediated by Piwi (10). Here, we revealed that nuclear Piwi loss affects the chromatin state of only a limited number of targets in the genome and we provide the data, indicating the existence of a mechanism of Piwi-mediated transcriptional silencing of transposons independent of H3K9me3 methylation and HP1a binding. We also demonstrate preferential Piwi-dependent repression of those transposons that display chromatin features compatible with a transcriptionally active state.

## MATERIALS AND METHODS

### ChIP-seq

Ovaries of 1–5 day old transheterozygous piwi^Nt^/piwi^2^ flies and their heterozygous siblings (piwi/+) were used. Two replicates of each ChIP-seq experiment were done. Сhromatin IP assay was performed as described previously (7), using polyclonal rabbit anti-HP1 (PRB-291C Covance innovative) and polyclonal rabbit antibodies (Upstate): Anti-H3K4me2 (#07–030) and Anti-H3K9me3 (#07–523). The quality of DNA precipitates was checked by real-time quantitative PCR before sequencing. For this we amplified intergenic 60D region, rp49 gene and HeT-A transposon using primers indicated previously (10). Samples containing 10–30 ng of precipitated DNA were used for sequencing. Input IP samples for each genotype were prepared and sequenced in parallel and the obtained data were used further for the normalization and calculation of enrichment levels. Illumina HiSeq platform was used for sequencing. All deep sequencing data are deposited with the Gene Expression Omnibus (GEO) under the accession number GSE56347.

### Accession Number

GSE56347

### Bioinformatic analysis

ChIP-seq reads were processed and trimmed. The numbers of reads per sample vary from approximately 14 million for H3K4me2 precipitate to 3 million for input samples. FASTQ files were assembled to reference (*D. melanogaster* genome Release 5, dm3) in CLC Genomics Workbench 3.5 application using its assembly algorithm. Inspection of resulting contigs reveals a high degree of similarity between two biological replicas, so we pooled sequencing data from two repeats for each sample type (HP1a, H3K4me2, H3K9me3 and input) and assembled to the reference dm3 genome. Mapping was performed either by mapping only uniquely positioned reads (single mapping, discarding non-specific matches) or mapping all the reads by assigning non-uniquely positioned reads to a random position in the genome (multi-mapping, random distribution function of CLC Genomics). The parameters of the assembler were: mismatch cost = 2, insertion cost = 3, deletion cost = 3, limit = 8. Ambiguity nucleotides were replaced by FASTA codes. Final genome coverage (in both genotypes is approximately the same) was HP1a sample—5×, H3K4me2—7.4×, H3K9me3—5.7×, input—3.5×. Coverage data for different regions were exported from CLC Genomics workbench into CSV files (chromosome position coverage) and used for subsequent analysis. Consensus sequences of transposable elements were obtained from Flybase (http://flybase.org/static_pages/downloads/FB2013_06/transposons/transposon_sequence_set.embl.txt.gz). piRNA cluster coordinates were taken from the piRNA bank http://pirnabank.ibab.ac.in and converted to Release 5 of *D. melanogaster* genome for subsequent analysis. For the analysis of dual-strand clusters we took the clusters, producing nearly equal amounts of piRNAs homologous to two complementary strands.

### ChIP-PCR

Unique insertions of blood and invader3 transposons on chromosome 2L were analyzed by quantitative ChIP-PCR using primers to transposon-genome junctions and genomic sequences flanking insertions. PCR product quantities were normalized to those of the input and relations to a fragment of an intergenic spacer in the 60D region were calculated. Following primers were used: GTGACTCTCACATCCCGAATC and CAGAACCGTTTCTGCTACTCGAAG for blood transposon-genome junction; TTTCCGTATCTGTGTGCTGT and TGGTCTGCGGATAATAATAC for adjacent genome region; AAACCGTTTGTAATACTTATTGCCT and ATTCATTTTTCGGCGTCCAC for invader3-genome junction; AATATAGTGTCTGGAAGTGTAAGGAAATGT and AAAAGGAAGTTCGCCGGTTG for adjacent genome region.

### Northern blot of snRNAs

Short RNAs (up to 200 bp) were extracted from ovaries of piwi^Nt^/piwi^2^ and piwi/+ 5 day old adult females using mirVana miRNA Isolation kit (Ambion) according to the manufacturer's protocol. Samples (1 μg RNA per lane) were loaded onto denaturing 15% PAAG gel, separated by electrophoresis, blotted onto Hybond NX membrane and fixed by UV (0.12J). Membranes were blocked in DIG Easy Hyb for 1 h at 42°C, hybridized to biotinylated probes in DIG Easy Hyb with 0.5 mg/ml yeast tRNA overnight at 42°C, washed 2× in 2×SSC with 0.1% SDS at room temperature and 2× in 0.1×SSC with 0.1% SDS at 45°C 10 min each. For staining, membranes were blocked in phosphate buffered saline with 0.5% SDS and 0.2% purified casein for 1 h at room temperature, washed in alkaline phosphatase (AP) buffer (0.1 M Tris pH 9.5, 0.1 M NaCl) and incubated 1 h in AP buffer with Streptavidin-AP (Invitrogen), dilution 1:1000. Then, membranes were washed in AP buffer (3 × 5 min) and developed in AP buffer with NBT/BCIP mix (20 μl/ml). For detection of U5 snRNA we used a mix of the following 5’-biotinylated hybridization probes: tggcccagttaccaaagtcgccgggcactacaaaataata, ttttagactcattagagtgttcctctccacggaaatcttt, agtaaaaggcgaaagatttattcgacaattgaagagaaaccagag. The probes were diluted in DIG Easy Hyb with 0.5 mg/ml yeast tRNA at 10 pm/ml concentration and denatured at 80°C for 5 min before usage. The 2S rRNA was used as a loading control. Hybridization probe for 2S rRNA was 5’Biot-TACAACCCTCAACCATATGTAGTCCAAGCA.

### RT-PCR

RNA samples were extracted from piwi^Nt^/piwi^2^ and piwi/+ ovaries of 2–3 day old females using Trizol RNA isolation protocol and reverse transcribed using Superscript II (Invitrogen) and random hexamer mix as primers. cDNAs were purified and quantitative real-time PCR was performed using primers for U1, U5 and U7 snRNAs and primers for housekeeping gene RpL32 as a load control. Three independent biological replica were analyzed. Each PCR was performed at least three times.

Primers for qPCR: U1 snRNA ATCACGAAGGCGGTTCCT and ACCAAAAATTACACGCACGAG; U5 snRNA CTGGTTTCTCTTCAATTGTC and TCGGGGCTCTAAGCAAA; U7 snRNA GAAATTTGTCTTGGTGGG and AACGGGAACACTCAATG; RpL32 ATGACCATCCGCCCAGCATAC and GCTTAGCATATCGATCCGACTGG.

### Microarray analysis

Total RNA samples were extracted from ovaries of piwi^Nt^/piwi^2^ and piwi/+ 2–3 day old females. Total RNA (30–40 μg) was isolated from 30 to 50 pairs of ovaries using Qiagen RNAeasy Mini Kit according to the manufacturer's instructions. RNA was immediately used for indirect labeling reaction. cDNA samples were prepared by reverse transcription, IVT-amplified and labeled with Cy3 or Cy5. Mix of differently labeled RNAs was hybridized to Oligo14Kv2 microarray slides (CDMC), washed, scanned and treated in GenePix 6 (Molecular Devices) and subsequently in FlexArray 1.6.2 (McGill University and Génome Québec Innovation Centre). Three biological replicas (one sample dye-swapped) were produced and analyzed. The microarray data were submitted to GEO (Series GSE40768). Detailed procedure of microarray treatment is described in GEO submission.

## RESULTS

### Transposons with a higher level of H3K4me2 mark are prone to Piwi-mediated repression

To reveal the chromatin changes due to the loss of Piwi silencing function, we estimated the levels of H3K4me2, H3K9me3 and HP1a marks by ChIP combined with deep sequencing (ChIP-seq) for the ovaries from transheterozygous piwi^Nt^/piwi^2^ flies (further designated as piwi^Nt^) and their heterozygous siblings (piwi/+). The use of piwi^Nt^ mutants lacking the Piwi NLS signal (10) for this analysis is advantageous for several reasons. First, the piwi^Nt^ mutation abolishes the silencing function of the Piwi protein in both germline and somatic ovarian cells since the early stages of development, whereas ovarian Gal4 drivers used for RNAi knockdowns exhibit cell-specific and stage-specific expression patterns. Second, in contrast to piwi null mutants and piwi knockdowns in somatic cells, ovaries of piwi^Nt^ flies show almost wild-type morphology, which allows to avoid the influence of indirect effects of Piwi depletion on chromatin. Third, we analyze specifically the lack of nuclear Piwi function, while the processes connected with its possible functioning in the cytoplasm remain undisturbed.

**Figure 1.**
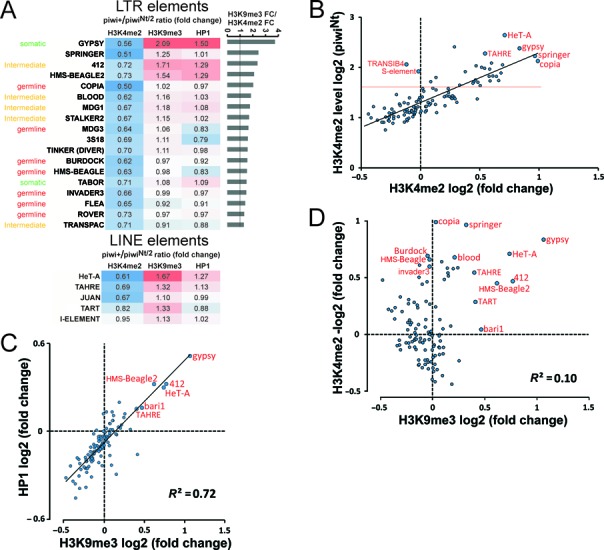
Analysis of histone modifications and HP1a occupancy over transposons upon the loss of nuclear Piwi. (**A**) List of LTR and LINE retrotransposons with the most highly changed chromatin state in piwi^Nt^/piwi^2^ mutant ovaries as compared with piwi/+ ones. The presented values are fold changes (FC) of histone marks H3K4me2 (transcription) and H3K9me3 and HP1a (heterochromatin) abundance in piwi/+ ovaries (control) divided by piwi^Nt^/piwi^2^ normalized to input. Transposons are ranked according to the decrease of the ratio of H3K9me3 FC to H3K4me2 FC. Depending on the transcription pattern, LTR elements are divided into germline-biased, intermediate, soma-biased (according to Malone *et al.* (29)) and undetermined. LINE transposons are thought to be expressed in the germline. (**B**) Scatter plot of input-normalized enrichment levels (log2) of H3K4me2 in the mutant piwi^Nt^/piwi^2^ ovaries (Y-axis) versus the FC value of H3K4me2 caused by the mutation (X-axis) for all transposons (*n* = 115). Selected transposons indicated by bigger dots are signed. The red line distinguishes transposons with a high H3K4me2 enrichment level. (**C**) FC of transposon H3K9me3 occupancies in piwi^Nt^/piwi^2^ mutants correlate positively with changes of HP1a level (*R*^2^ = 0.72). Selected transposons with significantly reduced levels of H3K9me3 and HP1a are indicated. (**D**) No inverse relationship is detected between H3K4me2 and H3K9me3 FC (*R*^2^ = 0.10) due to the piwi^Nt^/piwi^2^ mutation. A group of transposons (copia, springer, burdock, blood and others) display an increase of the H3K4me2 level, but no significant changes of the H3K9me3 level.

The H3K9me3 and HP1a marks are characteristic for heterochromatin and are also associated with a lot of sites in euchromatin, in particular with some transposon insertions and some protein-coding genes (23,26). The H3K4me2 mark is a reliable indicator of transcriptional activity, since H3K4me2 enrichment is almost equivalent to Pol II occupancy for different chromatin states in the *D. melanogaster* genome, including states of promoters, gene bodies, intronic and intergenic regions and silenced chromatin (27). The genome of *D. melanogaster* harbors diverse families of transposable and other repetitive elements that make up more than 70% of the annotated heterochromatin and 7% of euchromatin (28,29). Among the analyzed 120 transposon families in piwi^Nt^ ovaries, we observed a significant increase (over 20%) of H3K4me2 level for 19 families of long terminal repeats (LTR) containing elements and for three LINE-type retrotransposons (Supplementary Figure S1). LTR-elements belong to the germline- or soma-biased and intermediate elements, depending on their transcription pattern, whereas LINE transposons are thought to be expressed in the germline (29). Note that ChIP analysis of whole ovaries reflects the chromatin states in both the germline nurse cells that are highly polyploid and somatic cells that are more numerous, but have less chromosome ploidy (30–32). Among the LTR transposons for which the piwi^Nt^ mutation leads to the strongest alterations of the studied marks, there were representatives of all the types, including the somatic gypsy transposon, intermediate (412, blood, mdg1, stalker2) and germline-biased ones (copia, mdg3 and others) (Figure 1A). Among LINEs, the most notable increase of H3K4me2 was detected for telomeric LINEs (HeT-A, TAHRE) and the non-telomeric Juan transposon (Figure 1A). However, most LTR- and LINE-type transposons do not display significant changes in chromatin mark occupancy and no noticeable alterations were detected for DNA transposons (Supplementary Figure S1). It is unknown why Piwi regulates the chromatin state of only a limited number of transposons. We found that this specificity can be simply explained by the level of the target transcription. By dividing the amounts of H3K4me2 ChIP-seq reads to the input read numbers for each family of transposons, we calculated H3K4me2 enrichment levels that indicate the relative transcription activity of a given transposon type in the genome, irrespectively of its length and copy number. The majority of transposons, the chromatin states of which are not regulated by Piwi, show low H3K4me2 enrichments (Figure 1B). The notable exceptions are Piwi-insensitive DNA-transposons S-element and TRANSIB4 with a comparatively high H3K4me2 enrichment level (Figure 1B). Importantly, the insensitivity of chromatin of most transposons to Piwi cannot be explained by the lack of corresponding piRNAs loaded in a complex with Piwi. Significant amounts of piRNAs complementary to Piwi-insensitive transposons (e.g. Max-, X-, F-, stalker elements) have been immunoprecipitated with Piwi (33). Although we cannot exclude that these elements are represented by predominantly defective copies unable to transcribe in the studied fly stock, in other stocks the presence of full-length copies of these transposons was shown (34). The aub and ago-3 mutations affecting the cytoplasmic piRNA machinery were shown to upregulate some of these elements (35), indicating that their transcripts have the capacity to be accumulated in case of piRNA pathway disruption. Thus, we propose that Piwi-piRNA complexes are capable of recognizing transcripts of most transposons, but induce transcriptional silencing of only those targets, which have a high H3K4me2 level (transcription ability), whereas weakly transcribed transposons avoid Piwi-dependent repression at the chromatin level. The inherent level of transposon transcription may depend on their promoter strengths and localization in the genome. Of note, among the transposons with the highest H3K4me2 enrichment levels, we found telomeric elements (HeT-A, TAHRE and TART) that are known to be attached to chromosome ends and play an important role in telomere length maintenance (36).

### Transcriptional silencing by Piwi is not necessarily coupled with acquisition of H3K9me3 and HP1a marks

We found that the absence of nuclear Piwi significantly reduces the H3K9me3 and HP1a levels only for several transposon types, including LTR elements (e.g. gypsy, 412, HMS-Beagle2, mdg1) and germline-expressed telomeric LINEs (e.g. HeT-A, TAHRE, TART). Many other transposons show small fluctuations of the level of these marks in piwi^Nt^ ovaries compared with the control. When estimated for the bulk of transposons, H3K9me3 changes correlate highly with the shifts of HP1a level (*R*^2^ coefficient 0.72) (Figure 1C). This result supports that the H3K9me3 mark serves as a binding site to recruit HP1a in the course of transposon silencing in ovaries. We also observed that HP1a enrichment levels in both piwi/+ and piwi^Nt^ ovaries show a tight dependence on the corresponding H3K9me3 levels (Supplementary Figure S2). However, telomeric transposons (notably, TART) do not obey this correlation (Supplementary Figure S2). These transposons display a highly pronounced HP1a enrichment that can be explained by the known direct HP1a binding to telomeric DNA independently of H3K9 methylation (37).

**Figure 2.**
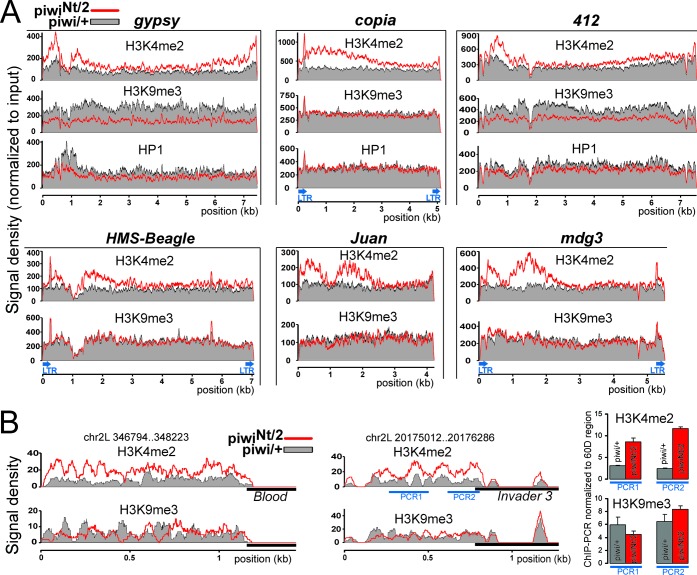
(**A**) Densities of the H3K4me2, H3K9me3 and HP1a for particular transposon consensus sequences (ChIP-seq reads) in the control (piwi/+) and piwi^Nt^/piwi^2^ samples (black/gray and red curves, respectively). Total read number was multiplied by the coefficient (input piwi/+/ input piwi^Nt^/piwi^2^) reflecting the difference in transposon abundance between genotypes (for the majority of transposons this coefficient is about 1.0). Examples of transposons which display both an increase of H3K4me2 and a decrease of H3K9me3 and HP1a (gypsy and 412) in piwi^Nt^ mutants, and those demonstrating only H3K4me2 changes (copia, HMS-Beagle, Juan and mdg3) are shown. H3K4me2 level increases mainly in the certain regions that correspond to LTRs, promoters and UTR regions. (**B**) Examples of H3K4me2 and H3K9me3 mark distributions in chromatin of genome regions adjacent to euchromatic transposon insertions. The regions adjacent to blood and invader3 insertions on chromosome 2L are shown. Transposon bodies are shown by black rectangles. The regions not covered by ChIP-seq reads are represented by non-unique sequences. Results of ChIP-PCR using primers to invader3-genome junction (PCR2) and genomic sequence flanking insertion (PCR1) are shown on the right.

For a lot of elements the increase in enrichment of the transcriptional H3K4me2 mark was not accompanied by a reduction of H3K9me3 level (Figure 1D). Notably, a group of non-telomeric germline retrotransposons (copia, HMS-Beagle, burdock, blood, invader3 and others) with a changed H3K4me2 level displayed slight or no changes of the H3K9me3 mark in the mutant ovaries (Figures 1D and 2A and Supplementary Figure S3). Similar results for copia and HMS-Beagle transposons have been obtained previously by ChIP-PCR (10). When estimated for all transposons, no inverse relationship was observed between the alterations of H3K4me2 and H3K9me3 levels due to the piwi^Nt^ mutation (*R*^2^ coefficient 0.1) (Figure 1D). Note that the strength of the observed changes of the H3K9me3 level for gypsy, 412, mdg1 and HeT-A transposons (Figure 2A and Supplementary Figure S3) is quantitatively very similar to those reported upon piwi knockdown in the ovarian somatic cells and ovaries (11,13). Therefore, the revealed low or no effect of the piwi^Nt^ mutation on H3K9me3 and HP1a levels of a group of Piwi-regulated transposons is unlikely to be attributed to the peculiarities of the experimental system used and rather indicates the existence of different types of transposon regulation.

To verify the observed effects of the Piwi mutation on the H3K4me2 level without changes of the H3K9me3 level, we analyzed the chromatin state in the regions adjacent to individual transposon insertions. By mapping unique ChIP-seq reads to the genome, we revealed euchromatic sites, where the most prominent increase of H3K4me2 abundance occurred due to the piwi^Nt^ mutation (genome coordinates are indicated in Supplementary Table S1). Some of them were found to be adjacent to transposon insertions, which were revealed by the presence of transposon-genomic junction reads and confirmed by PCR-analysis (Supplementary Figure S4). For example, strong elevations of H3K4me2 were observed for genomic sequences flanking blood and invader3 transposon insertions on chromosome 2L (Figure 2B). These elements belong to the group of germline-dominant transposons, for which the H3K4me2 mark increases, but no changes of the H3K9me3 mark were revealed in piwi^Nt^ mutants (Figure 1D). Consistent with this, no decrease of the H3K9me3 level was observed in regions flanking their insertions in piwi^Nt^ ovaries compared to the control that was confirmed by ChIP-PCR (Figure 2B). Note that the level of the H3K9me3 mark in the region flanking invader3 insertion was higher than the average genome coverage for this modification, indicating moderate H3K9me3 spreading from transposon to adjacent chromatin.

**Figure 3.**
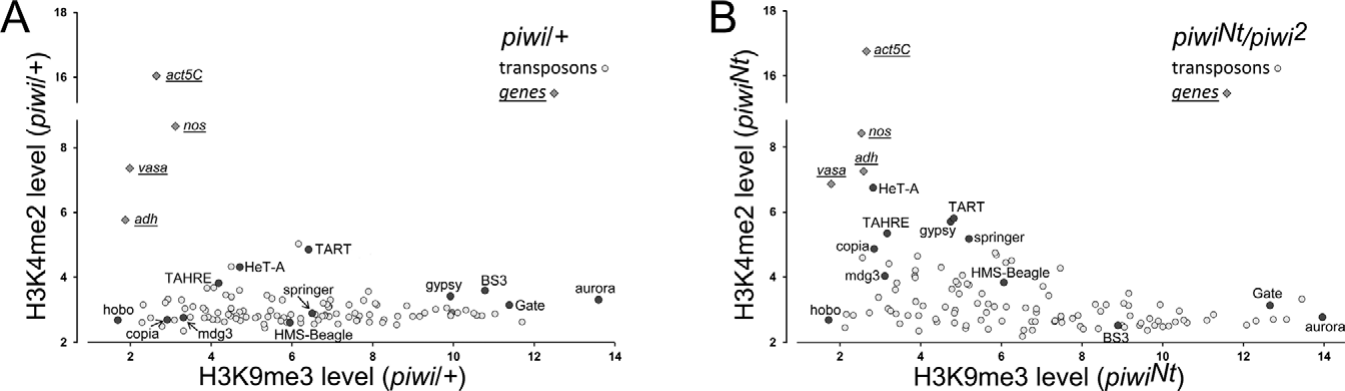
Input-normalized enrichment levels of H3K4me2 and H3K9me3 marks for different transposon families in piwi/+ (**A**) and piwi^Nt^/piwi^2^ ovaries (**B**). Selected transposons indicated by dark circles are signed. Enrichments of these chromatin marks in several protein-coding genes expressed in ovaries (adh, nanos, actin5C, vasa) are shown in rhombs.

Next, we examined whether the presence of the H3K9me3 mark impedes the H3K4me2 modification or these marks can coexist in transposon bodies. It is known that the H3K9me3 mark and HP1a are not attributed exclusively to the repressive chromatin state and have been reported to be required for transcription activation of some genes located in both eu- and heterochromatic parts of the genome (23,38–42). Our ChIP-seq data shows that transposons in ovaries have different H3K9me3 enrichment levels that vary 8-fold between different transposon families (Figure 3). For example, aurora and Gate elements, known to be located in heterochromatin (43,44), are highly enriched in the H3K9me3 mark (over 10-fold enrichment on Figure 3), whereas basically euchromatic copia and mdg3 transposons (34,45) harbor a comparatively low H3K9me3 enrichment (Figure 3A). In contrast, the enrichment level of the H3K4me2 mark is low for most transposons and differs only slightly between the elements in the control piwi/+ ovaries (Figure 3A). The highest H3K4me2 enrichment level was revealed for telomeric (HeT-A, TAHRE and TART) and a few DNA elements, as mentioned above. We failed to observe any correlation between H3K9me3/HP1 and H3K4me2 enrichments (Pearson coefficient 0.01), comparing these values for transposons in the piwi/+ ovaries. Thus, the wild-type level of H3K4me2 does not necessarily depend on whether copies of a particular transposon contain a high or a low level of heterochromatic marks.

In the piwi^Nt^ mutant, most transposons retain H3K9me3/HP1a enrichment, which can be clearly seen by comparing H3K9me3 levels of transposons with those of protein-coding genes (Figure 3B). Thus, the heterochromatic state for transposon bodies in ovaries is mostly produced by Piwi-independent pathways. Among the germline-expressed elements with severely changed H3K4me2 level in piwi^Nt^ mutants are examples of transposons enriched by H3K9me3 to a different extent. Telomeric transposons (HeT-A, TART, TAHRE) have a medium H3K9me3 level in piwi/+ ovaries (4–7-fold enrichment in Figure 3A) that is reduced to a low level by the piwi^Nt^ mutation (Figure 3B). HMS-Beagle and invador3 retain an unchanged medium H3K9me3 level in spite of transcriptional activation, whereas copia and mdg3 harbor a low enrichment level of H3K9me3 mark (comparable with active genes) in both piwi/+ and piwi^Nt^ ovaries (Figure 3). However, no transposons simultaneously display high levels of H3K9me3/HP1a and H3K4me2 marks in piwi^Nt^ ovaries, suggesting that transposons highly enriched by the H3K9me3 mark are repressed by the heterochromatic environment in the absence of Piwi-mediated silencing (Figure 3B). In piwi^Nt^ ovaries H3K9me3 and H3K4me2 enrichment levels show a moderate inverse relationship (Pearson coefficient −0.4). Taken together, these data suggest that some transposons can be transcribed in spite of the presence of a medium enrichment level of heterochromatic marks (several times higher than the level of these marks in euchromatic protein coding genes), whereas a very high H3K9me3 level is always associated with transposon repression. Interestingly, it has been shown that in somatic tissues different transposons within euchromatic regions display complex chromatin signatures, including enrichment in both heterochromatic and active marks (26).

### Chromatin state of dual-strand piRNA clusters is resistant to nuclear Piwi loss

piRNA-producing clusters composed of defective transposon copies encode piRNAs either on both genomic strands (dual-strand clusters) or predominantly on one strand (uni-strand clusters—e.g. flamenco) (15). Transcripts of the dual-strand clusters might be potentially recognized by Piwi complexes guided by piRNAs derived from the opposite RNA strand of the cluster. It has been shown that piRNA clusters require the H3K9me3 modification produced by the methyltransferase SetDB1 (Egg) for their transcription (46). To check whether nuclear Piwi is involved in the regulation of chromatin state of dual-strand piRNA clusters, we mapped unique ChIP-seq reads to the clusters, which produce practically equal amounts of piRNAs related to complementary strands (according to the databases). No significant changes of H3K4me2, H3K9me3 and HP1a occupancy in piwi^Nt^ ovaries were found for most of the analyzed clusters, including cluster1 (42AB) (Supplementary Figure S5E–G). Within the piRNA clusters on chromosome 4 we observed areas with high H3K9me3 occupancy that was slightly reduced in piwi^Nt^ mutants, but not accompanied by an increase of H3K4me2 level (Supplementary Figure S5A–D). We also observed highly pronounced discrete HP1a peaks within the chromosome 4 clusters (Supplementary Figure S5A–D). In contrast to HP1a distribution on other chromosomes, these HP1a peaks were not accompanied by H3K9me3 peaks, but they strongly coincided with the promoters of genes located within the clusters and were situated adjacent to H3K4me2 peaks in the gene transcribed sequences (Supplementary Figure S5A–D). Both the HP1a and the adjacent H3K4me2 peaks remained unchanged in the piwi^Nt^ mutant (Supplementary Figure S5A–D). The observed chromatin state of these clusters is in accord with the reports that HP1a binds to promoters of active genes in chromosome 4 chromatin independently of the H3K9me mark (47).

Importantly, we did not find H3K4me2 coverage that would be significantly above background for the analyzed piRNA clusters with the exception of the areas of protein-coding genes located within the clusters (Supplementary Figure S5), indicating that these clusters have a low transcriptional rate. Thus, we suggest that dual-strand piRNA clusters may avoid Piwi-mediated repression similarly to the weakly transcribed transposons.

### Loss of nuclear Piwi exerts no significant effect on expression of protein-coding genes, but increases snRNA abundance

**Figure 4.**
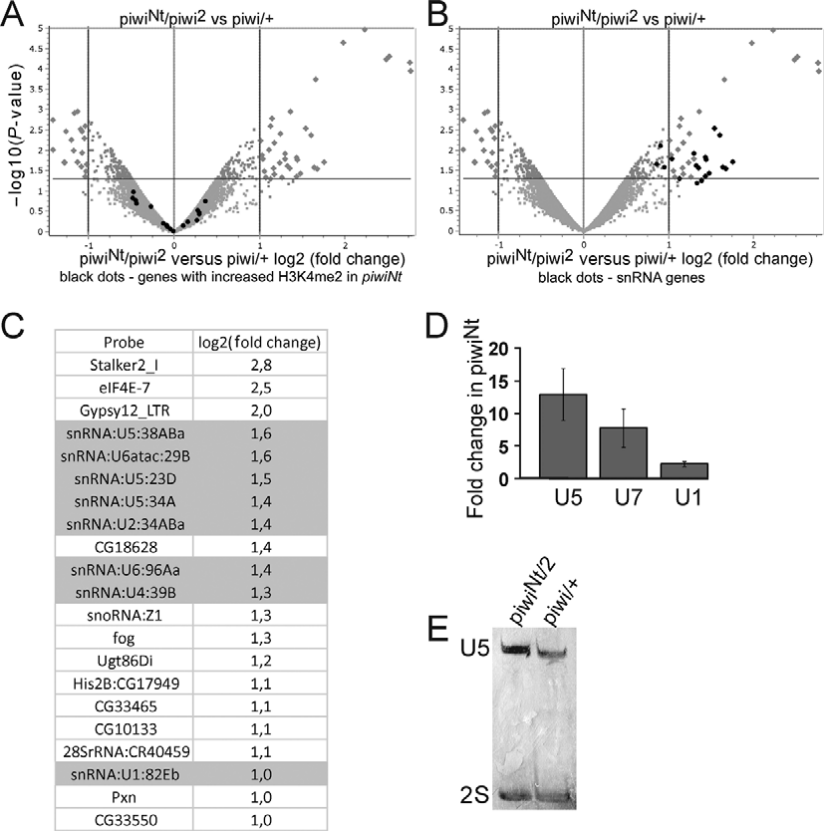
Effect of nuclear Piwi elimination on gene expression. (**A**) Volcano plot represents gene expression in piwi^Nt^/piwi^2^ ovaries versus the control (piwi/+) based on four microarrays. Bigger dots indicate genes that display both large-magnitude fold-changes (X-axis) and high statistical significance (−log10 of *P*-value, Y-axis). Black dots correspond to genes containing regions with significantly increased H3K4me2 level in piwi^Nt^/piwi^2^ ovaries according to ChIP-seq. (**B**) Volcano plot as in A with black dots indicating snRNA probes. (**C**) List of micro-array probes displaying the highest upregulation in piwi^Nt^/piwi^2^ mutants. snRNA probes are highlighted. The used microarray platform included a few probes corresponding to transposons (Stalker 2, gypsy 12) and these probes fell to the top of the list. (**D**) RT-PCR analysis of U1, U5 and U7 snRNAs in piwi^Nt^/piwi^2^ ovaries. (**E**) Northern blot demonstrates increase of U5 snRNA abundance caused by the piwi^Nt^ mutation. 2S rRNA was used as a loading control.

Reported data concerning the influence of the piRNA pathway and the Piwi protein on host protein coding gene expression are contradictory (11,13,14). To identify the targets of Piwi beyond transposons, we performed microarray-based expression profiling of piwi^Nt^/piwi^2^ mutant ovaries. No appreciable alterations in the abundances of the majority of unique transcripts were observed, whereas the expression of a minor fraction of genes was changed (Figure 4A). The observed upregulation of these genes in piwi^Nt^ ovaries is unlikely to be related to Piwi-mediated chromatin repression, because we have not detected any alterations of their chromatin state (data not shown). The altered expression of these genes can be caused by the existence of additional functions of the Piwi protein in post-transcriptional RNA regulation or by indirect effects. Nevertheless, ChIP-seq revealed a number of unique euchromatic sites with a significantly increased level of H3K4me2 due to the piwi^Nt^ mutation (Supplementary Table S1). Some of these sites were located close to transposon insertions adjacent to some genes. However, the bleeding of this mark usually did not exceed several hundreds bp, indicating short-distance spreading of Piwi-regulated chromatin state. We found that genes containing these H3K4me2 enriched peaks were not upregulated, judging by the microarray analysis of mRNA abundance (Figure 4A). Thus, even if Piwi-mediated transposon repression spread to neighboring genes, it did not affect the production of corresponding mRNAs. Interestingly, a noticeable increase of small nuclear spliceosomal RNAs (snRNAs) abundances, especially of U2, U5 and U6, was observed in piwi^Nt^ ovaries on the microarray (Figure 4B and C). This effect was confirmed by real-time PCR and northern blotting (Figure 4D and E). ChIP-seq data show no alterations in the levels of HP1a/H3K9me3 and H3K4me2 marks in the chromatin of snRNA genes, indicating nuclear Piwi regulation at the level of RNA stability. We suggest that Piwi may be involved in the post-transcriptional control of snRNA abundance in the nucleus that is realized by a mechanism distinct from piRNA-mediated repression, because no prominent fraction of small RNAs antisense to the snRNAs was found in the existing databases. It has been previously shown by IP experiments that Piwi may interact with numerous proteins participating in splicing (13). We cannot completely exclude the possibility that snRNA accumulation upon nuclear Piwi loss is an indirect effect, for example a stress response caused by transposon activation. However, no significant snRNA accumulation in some other piRNA pathway mutants was observed (data not shown), indicating that the effect is Piwi-specific.

## DISCUSSION

The use of an experimental system based on the cytoplasm-localized Piwi protein confirms the effect of Piwi on transposon transcription in ovaries of *D. melanogaster*. We found that Piwi-mediated transcriptional silencing is a highly selective process. Piwi is not implicated in global distribution of epigenetic marks and general heterochromatin formation, and does not affect significantly the chromatin state of the weakly transcribed transposons and piRNA clusters. In contrast to recently reported data (14) and in agreement with Toth *et al.* (13), our results indicate that Piwi-guided chromatin silencing does not influence, at least significantly, host protein-coding gene expression. We revealed that Piwi exerts more prominent repression and decrease of H3K4me2 level of those transposons that have a higher level of this modification and are prone to be transcribed, whereas weakly transcribed elements avoid Piwi-mediated silencing. This observation is in agreement with the generally accepted model that transcription of the target is required to initiate transcriptional silencing by the small RNA-guided complex (1).

We observe no correlation between the changes of transcription-associated H3K4me2 and heterochromatic H3K9me3/HP1a marks on transposons due to the loss of nuclear Piwi. Our results indicate that the Piwi-piRNA complex can repress transcription without the recruitment of H3K9me3 and HP1a, at least in the case of a number of non-telomeric transposons expressed in germline cells (Figure 2 and Supplementary Figure S3). The decrease of H3K9me3 and HP1a levels in the chromatin of some other transposons was observed due to the Piwi mutation in accord with previous reports (9–13). The alterations of the H3K9me3 and HP1a levels correlate strongly (Figure 1C), as well as the enrichments of these marks for most transposons in both the control and mutant ovaries (Supplementary Figure S2). These data support the view that HP1a recognizes H3K9me3 mark as a binding site in transposon sequences, rather than the model suggesting a direct association of HP1a with Piwi (48). Some Piwi-regulated elements (e.g. copia and mdg3) harbor a low level of heterochromatic marks in both the control and mutant ovaries. Some other transposons, such as HMS-Beagle, become transcriptionally active in the piwi^Nt^ mutant in spite of the remaining significant enrichment of H3K9me3 and HP1a in their bodies (Figure 3B). It has been shown that the H3K9me3 modification provided by Piwi is not sufficient per se for transposon silencing in ovarian somatic cell culture (11). Knockdown of a putative Piwi cofactor, the Maelstrom protein, leads to transcriptional activation of transposons similarly to the piwi knockdown, while the H3K9me3 mark is retained almost unchanged. The authors suggest that Maelstrom may act in silencing downstream of or in parallel to H3K9me3 methylation (11). Our data indicate that Piwi-mediated transcriptional silencing and H3K9me3 methylation are likely to be two parallel processes, whereas in case of some transposons the significance of the latter activity is not apparent. Taken together, it is likely that in addition to the reported triggering of H3K9me3 modification (10–13) the Piwi complex might repress the transcriptional machinery by an alternative, yet unknown mechanism. Interestingly, recently published data indicate that developmental silencing of a retrotransposon in mammals follows loss of activating marks rather than acquisition of conventional heterochromatic marks (49).

It remains poorly understood, whether heterochromatic marks themselves cause repression of transposon transcription in ovaries. HP1 is known to suppress genes through chromatin compaction that restricts the access of transcription factors (23,50) and it has been shown that HP1a knockdown leads to the derepression of several transposons in the germline (9). However, the association of HP1 with heterochromatin is known to be remarkably dynamic (51) and many cases of active transcription in HP1-enriched domains were found (23,38,40–42,52,53). Work on fission yeast S. pombe demonstrates that the HP1 homolog Swi6 protein represses heterochromatic genes through cotranscriptional RNA degradation, but not by a transcriptional block (54). Interaction of HP1 with RNA was also shown in mammals and *D. melanogaster* (39,55), suggesting that this mechanism may be conserved. We found that H3K9me3/HP1 enrichment for most transposons is resistant to the loss of Piwi, indicating the existence of Piwi-independent formation of their heterochromatic states in ovaries. Although some transposons are capable of transcribing despite a moderate presence of heterochromatic marks within their bodies, we did not observe transcriptional H3K4me2 mark enrichment for transposons, which harbor very high levels of H3K9me3/HP1 in piwi^Nt^ ovaries (Figure 3B). These findings suggest that Piwi-independent heterochromatinization has an impact on transposon silencing in ovaries, but it is not sufficient for complete repression of transposons in the absence of the piRNA pathway.

## SUPPLEMENTARY DATA

Supplementary Data are available at NAR Online.

SUPPLEMENTARY DATA
