# Supplementary material for: Impact of nuclear Piwi elimination on chromatin state in Drosophila melanogaster ovaries
Source: Nucleic Acids Res. 2014 Apr 29;42(10):6208–18. doi: 10.1093/nar/gku268 (PMC4041442; doi:10.1093/nar/gku268)
Supplement: SUPPLEMENTARY DATA [file supp_42_10_6208__index.html]

Impact of nuclear Piwi elimination on chromatin state in Drosophila melanogaster ovaries — Impact of nuclear Piwi elimination on chromatin state in Drosophila melanogaster ovaries — SUPPLEMENTARY DATA 

# Impact of nuclear Piwi elimination on chromatin state in *Drosophila melanogaster* ovaries

## SUPPLEMENTARY DATA

**Files in this Data Supplement:**

- SUPPLEMENTARY DATA
