# Supplementary material for: Impact of nuclear Piwi elimination on chromatin state in Drosophila melanogaster ovaries
Source: Nucleic Acids Res. 2014 Apr 29;42(10):6208–18. doi: 10.1093/nar/gku268 (PMC4041442; doi:10.1093/nar/gku268)
Supplement: SUPPLEMENTARY DATA [file supp_gku268_nar-00097-y-2014-File006.pdf]

Supplementary figures.

Figure S1 (addition to figure 1A). Heat map represents fold changes of histone marks H3K4me2 (transcriptional activation) and H3K9me3 and HP1a (heterochromatin) abundances in *piwi/+* (control) divided by those for *piwi*^{Nt}/*piwi*^{2} ovaries normalized to input. Transposable elements are ranked according to the decrease of the ratio of H3K9me3 fold changes to H3K4me2 fold changes (FC). Transposons are divided into 3 types: (i) LTR retrotransposons, (ii) LINE-like retrotransposons and (iii) and DNA transposons (class II). LTR-elements are divided into germline-biased, intermediate, soma-biased (according to Malone et al, 2009) and undetermined groups, depending on the expression patterns. The majority of elements do not show a significant change in either the histone marks density or HP1a occupancy.

LTR elements

piwi+/piwi$^{Nt/2}$ ratio (fold change)

		H3K4me2	H3K9me3	HP1
somatic	GYPSY	0.56	2.09	1.50
	SPRINGER	0.51	1.25	1.01
Intermediate	412	0.72	1.71	1.29
	HMS-BEAGLE2	0.73	1.54	1.29
germline	COPIA	0.50	1.02	0.97
Intermediate	BLOOD	0.62	1.16	1.03
Intermediate	MDG1	0.67	1.18	1.08
Intermediate	STALKER2	0.67	1.15	1.02
germline	MDG3	0.64	1.06	0.83
	3S18	0.69	1.11	0.79
	TINKER (DIVER)	0.70	1.11	0.98
germline	BURDOCK	0.62	0.97	0.92
germline	HMS-BEAGLE	0.63	0.98	0.83
somatic	TABOR	0.71	1.08	1.09
germline	INVADER3	0.66	0.99	0.97
germline	FLEA	0.65	0.92	0.91
germline	ROVER	0.73	0.97	0.97
Intermediate	TRANSPAC	0.71	0.91	0.88
germline	MICROPIA	0.74	0.93	0.90
Intermediate	STALKER4	0.85	1.02	0.96
	STALKER	0.83	0.98	0.94
germline	INVADER2	0.82	0.96	0.90
germline	297	0.97	1.07	1.01
Intermediate	GYPSY6	0.84	0.90	0.86
germline	GYPSY12	0.82	0.89	0.87
germline	17.6	0.85	0.91	0.85
germline	INVADER4	0.91	0.96	0.92
	OPUS	1.18	1.19	1.10
Intermediate	QUASIMODO	1.04	1.05	1.01
somatic	GTWIN	0.96	0.95	0.90
germline	ROO	1.08	1.08	1.06
Intermediate	GYPSY2	1.08	1.04	0.92
	McCLINTOCK	1.03	0.98	0.91
Intermediate	GYPSY4	1.07	1.01	0.97
germline	ACCORD	0.87	0.82	0.81
Intermediate	IDEFIX	0.92	0.86	0.85
germline	INVADER	1.08	1.01	0.96
	DM88	0.93	0.87	0.88
germline	GYPSY8	0.97	0.90	0.78
germline	1731	0.93	0.85	0.87
germline	MAX-ELEMENT	0.99	0.89	0.86
somatic	GYPSY10	1.14	1.02	1.01
germline	GATE	1.00	0.90	0.88
germline	DIVER2	0.87	0.76	0.76
	STALKER3	0.86	0.75	0.83
germline	ROOA LTR	1.04	0.89	0.90
	GYPSY11	0.91	0.78	0.75
	INVADER5	1.36	1.14	1.17
	FROGGER	1.18	0.99	1.10
Intermediate	GYPSY3	1.24	1.03	0.94
germline	ACCORD2	1.17	0.97	0.91
germline	CIRCE	1.08	0.89	0.92
somatic	ZAM	1.25	1.01	0.99
	AURORA	1.23	0.97	0.92
	GYPSY9	1.34	1.02	1.11
germline	GYPSY7	1.21	0.91	0.89

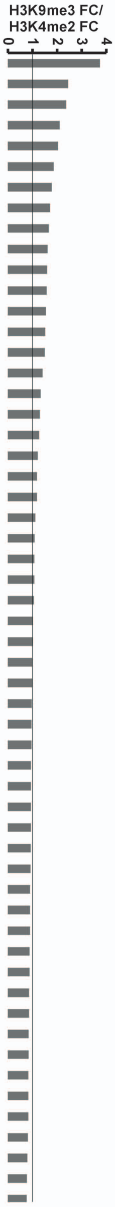

LINE elements

piwi+/piwi$^{Nt/2}$ ratio (fold change)

	H3K4me2	H3K9me3	HP1
HeT-A	0.61	1.67	1.27
TAHRE	0.69	1.32	1.13
JUAN	0.67	1.10	0.99
TART	0.82	1.33	0.88
I-ELEMENT	0.95	1.13	1.02
JOKEY	1.00	1.05	1.04
DOC	1.01	1.05	0.98
R1-2	0.85	0.78	0.70
F-ELEMENT	1.04	0.95	0.92
G4	1.05	0.94	0.92
DOC5 (PORTO-1)	1.05	0.92	0.89
Q-ELEMENT	0.97	0.85	0.82
R1A	1.03	0.89	0.87
DOC4	1.10	0.95	0.84
X-ELEMENT	1.02	0.86	0.82
RT1C	1.20	1.02	0.96
DOC3	1.05	0.87	0.83
JOCKEY2	1.29	1.07	1.11
RT1A	1.19	0.97	0.87
RT1B	1.10	0.90	0.85
G3	1.23	1.00	0.99
FW3	1.17	0.95	0.97
G5	1.05	0.85	0.86
G-ELEMENT	1.05	0.84	0.82
BAGGINS	1.08	0.86	0.80
G6	1.20	0.95	0.95
IVK	1.20	0.95	0.96
R1A1-ELEMENT	1.17	0.91	0.93
G2	1.04	0.80	0.86
BS	1.23	0.94	1.00
DOC2	1.20	0.92	0.89
G5A	1.32	1.00	0.97
R2	1.07	0.79	0.87
FW2	1.10	0.81	0.84

DNA elements*

piwi+/piwi$^{Nt/2}$ ratio (fold change)

	H3K4me2	H3K9me3	HP1
BARI1	0.97	1.38	1.13
TC1-2	0.91	1.05	1.04
TC3	0.81	0.85	0.85
TRANSIB1	0.83	0.85	0.74
LOOPER1	0.80	0.81	0.73
POGO	0.98	0.99	1.00
HOPPER2	0.72	0.72	0.78
S-ELEMENT	1.01	1.00	0.93
1360	0.99	0.97	0.95
HOBO	1.00	0.98	0.93
TRANSIB2	1.00	0.98	0.92
S2	0.94	0.91	0.99
TRANSIB4	1.09	1.05	0.88
TNFB	1.13	1.07	1.06
HB	1.13	1.05	0.99
TRANSIB3	0.94	0.86	0.82
HOPPER	1.10	1.00	0.96
TC1	1.02	0.92	0.92
INE-1	1.15	1.00	0.95
MARINER2	1.03	0.90	0.86
BARI2	1.07	0.80	0.78

* includes also ine-1 SINE element

Figure S2. Enrichment levels of H3K9me3 mark and HP1a in transposons in *piwi/+* and *piwi*^{Nt}/*piwi*^{2} ovaries. HP1a and H3K9me3 marks correlate strongly for most transposons with the exception of telomeric elements (*HeT-A*, *TAHRE*, and notably *TART*).

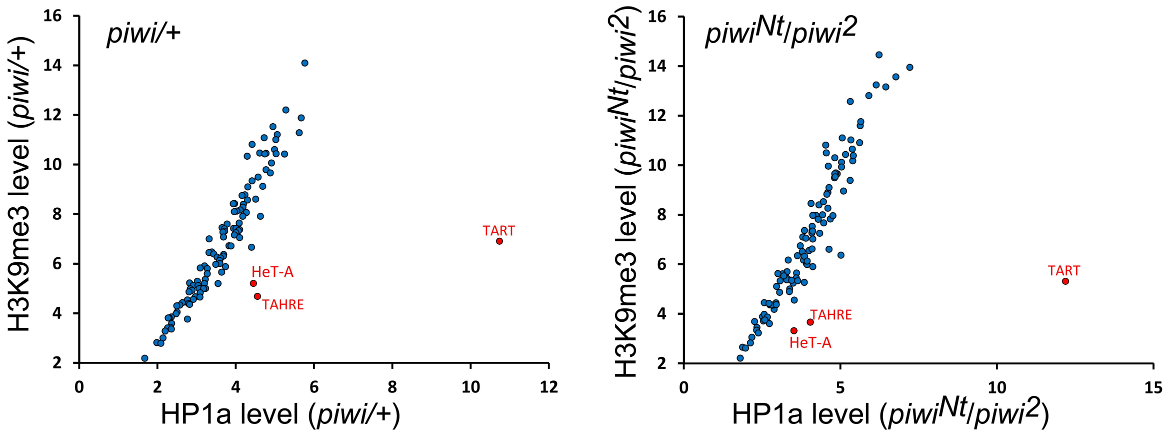

Figure S3 (addition to figure 2A). Densities of the H3K4me2 and H3K9me3 marks (ChIP-seq reads) for transposon consensus sequences in the control (*piwi*/+) and *piwi*^{Nt}/*piwi*^{2} samples (grey and red curves, respectively). Total read number was multiplied by the coefficient (input *piwi*/+/ input *piwi*^{Nt}/*piwi*^{2}) reflecting the difference in transposon abundance between genotypes (for the majority of transposons this coefficient equals to about 1.0).

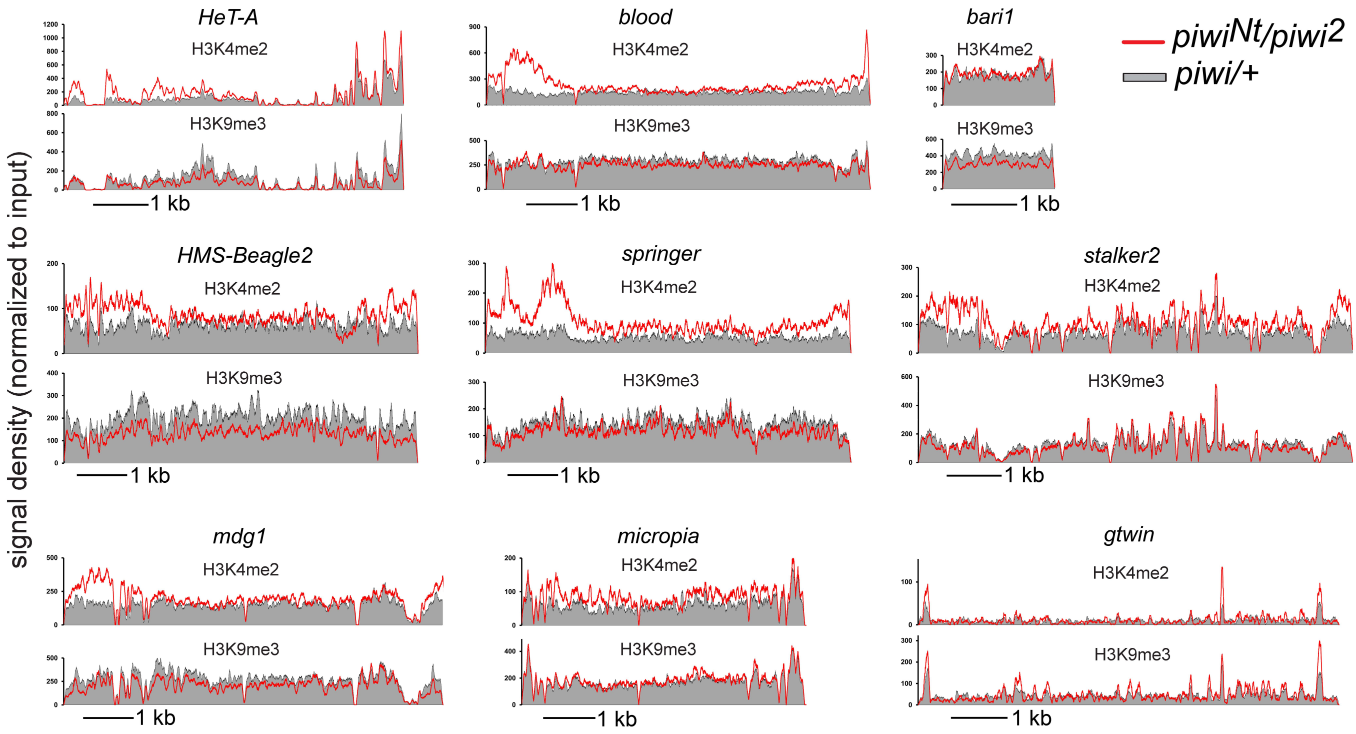

Figure S4. The presence of insertions of *blood* and *invader3* transposons on chromosome 2L was confirmed by PCR using primers to transposon-genome junctions and genomic sequences flanking insertions. Positions of primers and densities of H3K4me2 ChIP-seq reads are shown. Transposon bodies are indicated by purple rectangles.

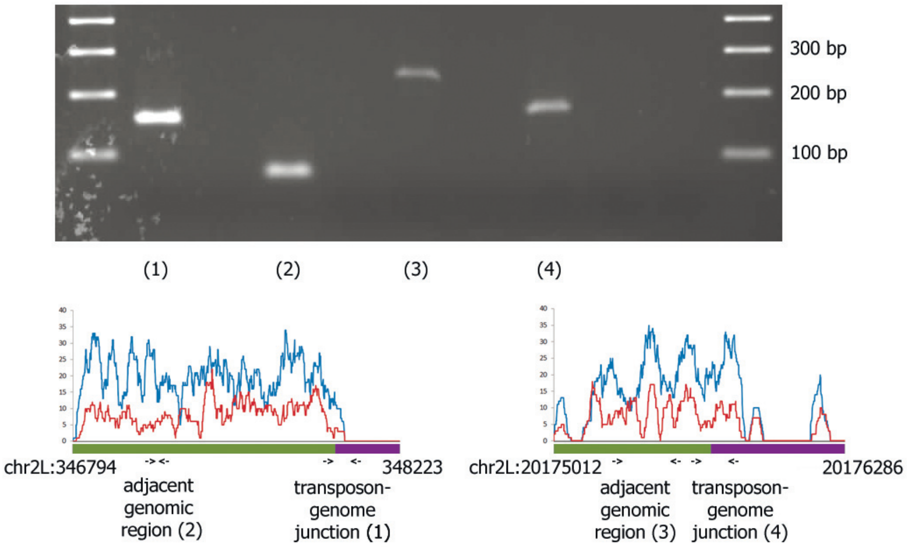

Figure S5. Chromatin state of a dual-strand piRNA clusters in *piwi*^{Nt}/*piwi*^{2} and *piwi*/+ ovaries. Profiles show distributions of H3K4me2, H3K9me3 and HP1a unique ChIP-seq reads smoothed by taking the moving average over ±100 points. The regions not covered by reads are represented by non-unique sequences. **(A-D)** Within the dual-strand piRNA clusters on chromosome 4 the highly pronounced HP1a peaks coincide with the promoters of genes located within the clusters and are adjacent to H3K4me2 peaks in gene transcribed sequences (indicated in blue as the HP1 H3K4me2 mark junction). The areas where the H3K9me3 level is reduced in *piwi*^{Nt}/*piwi*^{2} ovaries are highlighted by green (signed as H3K9me3 change). (**E, F)** Examples of clusters on chromosomes X and 3L. **(G)** Distribution of ChIP-seq reads over piRNA cluster 1 (42AB).

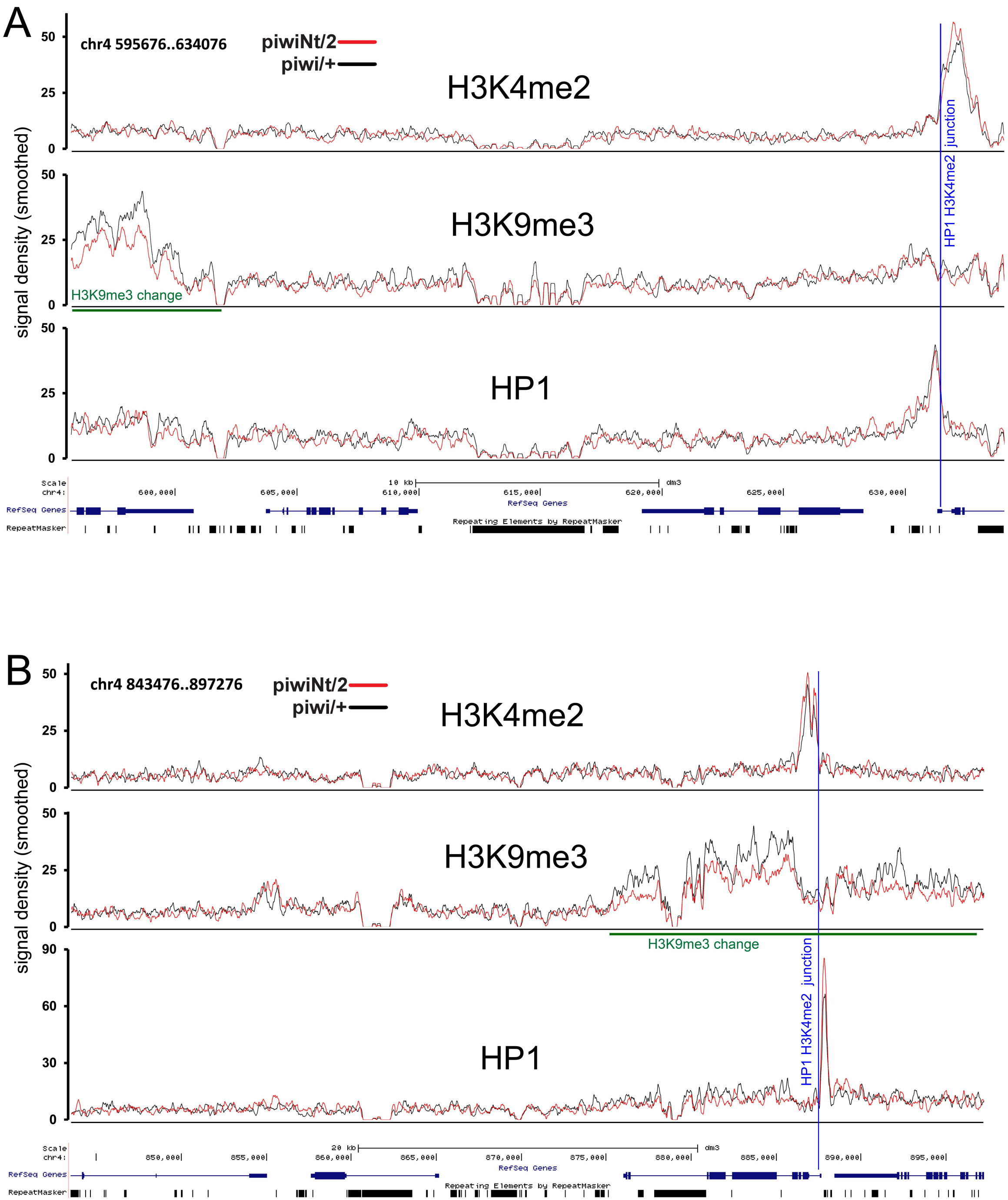

A
chr4 595676..634076
piwiNt/2
piwi/+
H3K4me2
H3K9me3
HP1
signal density (smoothed)
50
25
0
H3K9me3 change
HP1 H3K4me2 junction
Scale
chr4:
10 kb
dm3
600,000
605,000
610,000
615,000
620,000
625,000
630,000
RefSeq Genes
Repeating Elements by RepeatMasker
RepeatMasker
B
chr4 843476..897276
piwiNt/2
piwi/+
H3K4me2
H3K9me3
HP1
signal density (smoothed)
50
25
0
90
60
30
H3K9me3 change
HP1 H3K4me2 junction
Scale
chr4:
20 kb
dm3
850,000
855,000
860,000
865,000
870,000
875,000
880,000
885,000
890,000
895,000
RefSeq Genes
Repeating Elements by RepeatMasker
RepeatMasker

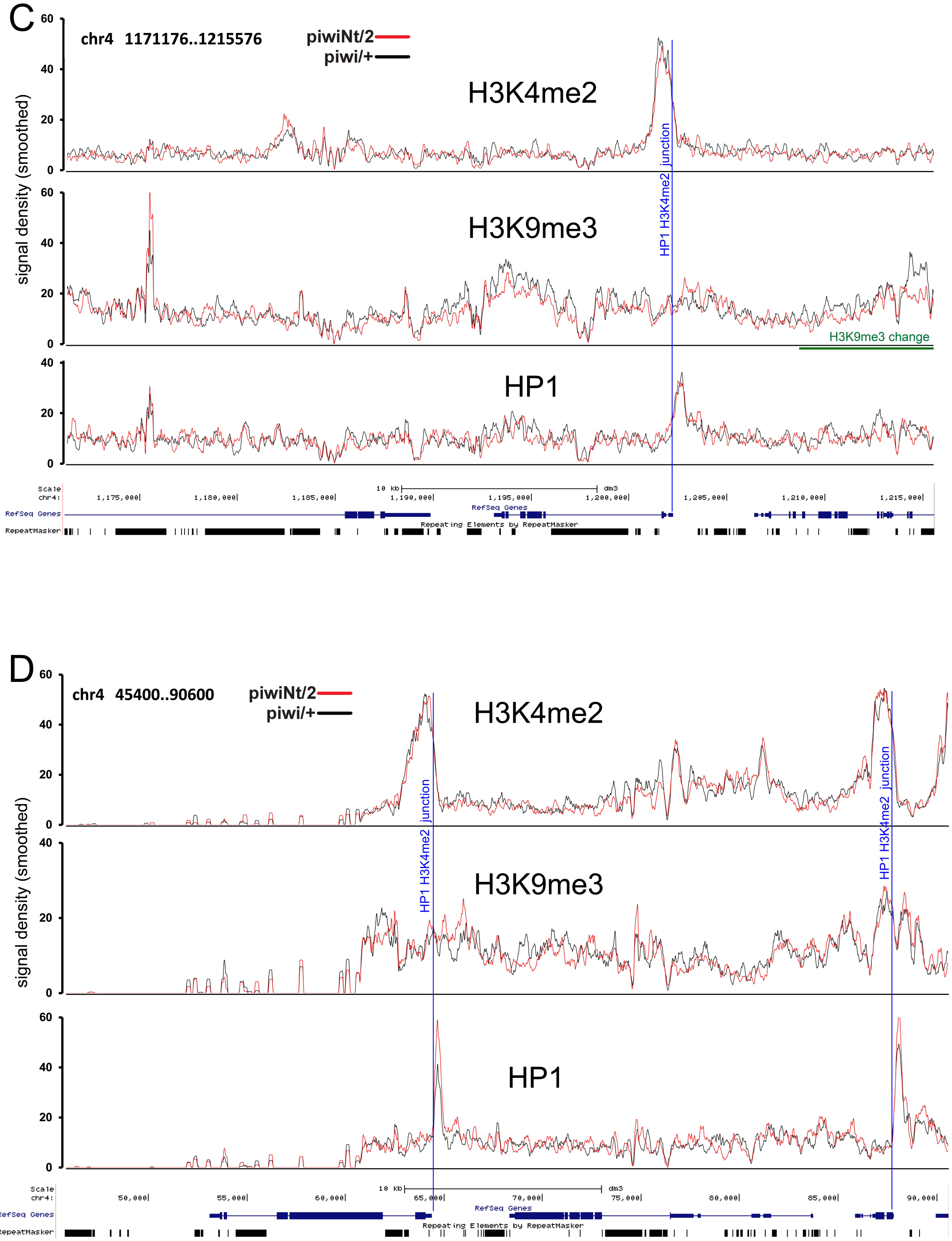
C
chr4 1171176..1215576
piwiNt/2
piwi/+
H3K4me2
H3K9me3
HP1
signal density (smoothed)
HP1 H3K4me2 junction
H3K9me3 change
Scale
chr4:
RefSeq Genes
Repeating Elements by RepeatMasker
RepeatMasker
D
chr4 45400..90600
piwiNt/2
piwi/+
H3K4me2
H3K9me3
HP1
signal density (smoothed)
HP1 H3K4me2 junction
Scale
chr4:
RefSeq Genes
Repeating Elements by RepeatMasker
RepeatMasker

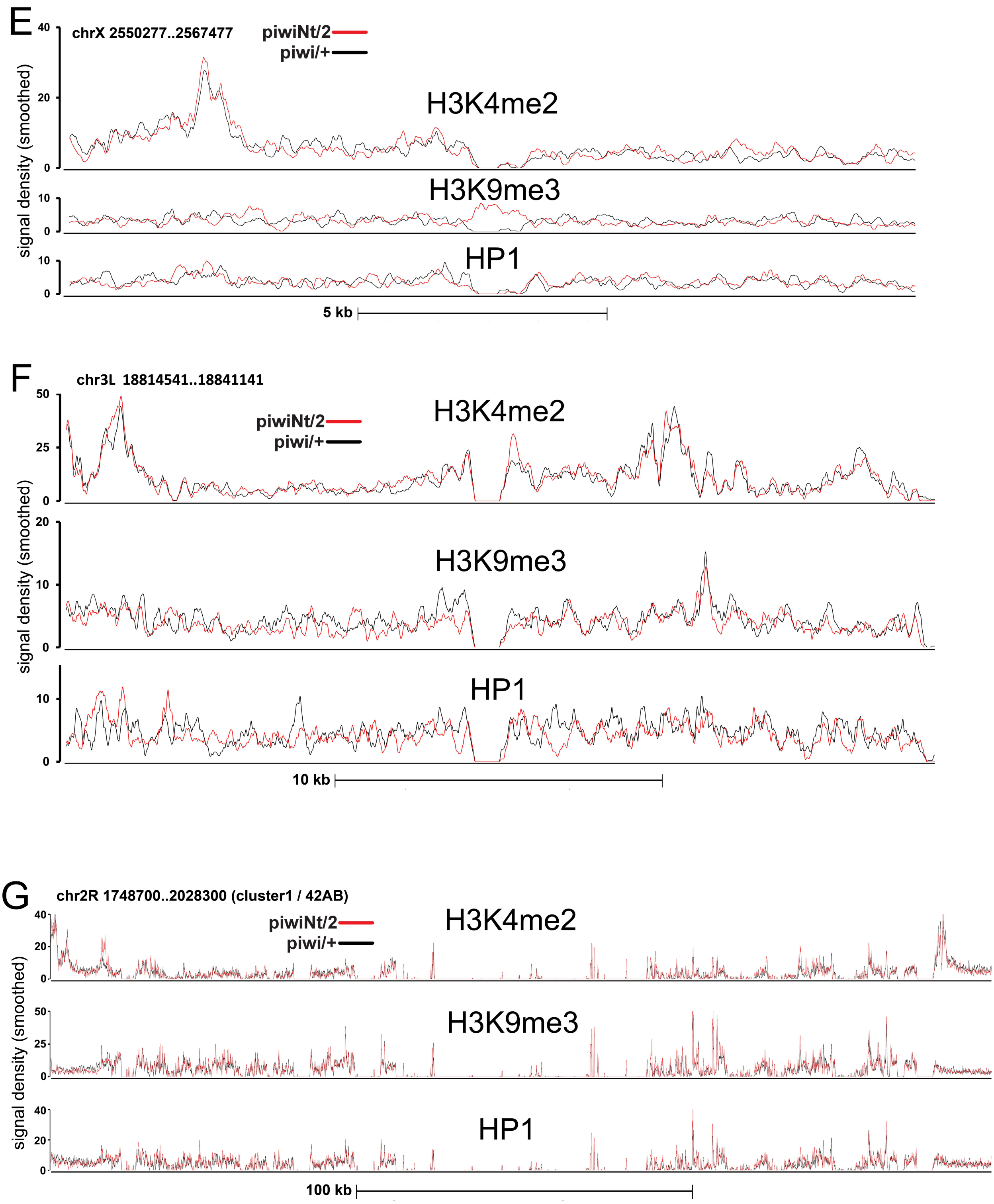
E
chrX 2550277..2567477
piwiNt/2
piwi/+
H3K4me2
H3K9me3
HP1
signal density (smoothed)
40
20
0
10
0
10
0
5 kb
F
chr3L 18814541..18841141
50
25
0
20
10
0
10
0
H3K4me2
H3K9me3
HP1
piwiNt/2
piwi/+
signal density (smoothed)
10 kb
G
chr2R 1748700..2028300 (cluster1 / 42AB)
piwiNt/2
piwi/+
H3K4me2
H3K9me3
HP1
40
20
0
50
25
0
40
20
0
signal density (smoothed)
100 kb

Supplementary tables

Table S1. Coordinates of genome regions with the increased H3K4me2 occupancy in *piwi*^{Nt}/*piwi*^{2} vs *piwi*/+ ovaries with the indication of adjacent genes.

Chr	Region	Length	FDR (%)	Normalized difference	**p-value**	5' gene	3' gene
2R	10509248..10509356	108	1,53	0,12	5,55E-17		mspo (50083)
3R	10480978..10481097	119	0,5	0,06	7,39E-13		sr (3435427)
2R	19242812..19242915	103	0,07	0,37	3,94E-12	CG30410	CG3500 (979)
chrX	8358197..8358281	84	2,66E-05	9,21E-03	4,35E-12	Es2 (779)	Sptr
3R	4169024..4169084	60	0,61	0,03	4,98E-12		sr (9747440)
chr4	1282655..1282743	88	4,16E-04	0,36	2,94E-10	Dyrk3 (48222)	
2R	6365684..6365748	64	0,65	0,13	8,38E-10		mspo (4193691)
chrX	17196596..17196648	52	0,85	0,39	1,17E-09	CG5445	B-H2 (11965)
chrX	1170196..1170273	77	1,07	0,11	1,20E-09		CG33080 (4432320)
2L	6098804..6098854	50	0,61	0,39	1,49E-09	Kr-h1 (2306)	CG9175
2L	19181566..19181632	66	0,57	0,29	1,52E-09	brat (8994)	CG17568
2R	3454534..3454575	41	3,22E-03	0,26	1,54E-09		mspo (7104864)
chrX	6697160..6697243	83	0,12	0,04	2,92E-09	shf (23468)	C3G
3L	5920941..5920996	55	1,91	0,1	4,98E-09		CG10426 (6349796)
3R	5868384..5868454	70	0,33	0,04	8,40E-09		sr (8048070)
2L	11107022..11107071	49	0,07	0,33	1,05E-08	l(2)gd1	CG6201 (3485)
3R	21154807..21154938	131	0,02	0,09	1,11E-08	CG10562 (6334)	CHKov1
3L	20394469..20394562	93	0,81	0,04	1,14E-08	CG33969 (7821)	trbl
2L	1728833..1728889	56	0,61	0,16	1,20E-08		CG11030 (3995768)
3R	21118506..21118552	46	1,95	0,1	1,40E-08	CG11893 (1544)	CG31098
2R	14293984..14294073	89	2,61E-05	0,02	2,61E-08	GstE6	GstE7 (366)
3L	8713039..8713071	32	4,62E-06	0,05	2,76E-08		CG10426 (3557721)
3R	19856965..19857033	68	0,06	0,39	2,95E-08	LSm3 (34145)	KrT95D
2L	73558..73592	34	6,12	0,05	3,35E-08		CG11030 (5651065)
chrX	6698260..6698349	89	0,48	0,04	3,35E-08	pod1	iav (9801)
3L	20190664..20190718	54	0,52	0,08	3,38E-08	CG42348 (33394)	CG32223
chrX	381717..381779	62	1,11	0,02	3,42E-08		CG33080 (5220814)
2R	14285953..14285996	43	1,80E-06	0,32	3,73E-08	GstE1	GstE2 (996)
3R	10522760..10522794	34	0,79	0,11	3,92E-08		sr (3393730)
2L	479999..480070	71	1,86	0,22	4,48E-08		CG11030 (5244587)
3L	2265640..2265668	28	0,07	2,68E-03	4,77E-08		CG10426 (10005124)
2L	16491097..16491165	68	0,83	0,12	5,88E-08	CG6488 (4991978)	beat-IIIc (698644)
2R	18784364..18784469	105	0,6	0,17	5,98E-08	nahoda	CG30187 (19895)
2R	11088291..11088331	40	0,94	0,11	6,01E-08	Sfp51E (7558)	CG8090
3R	25074376..25074443	67	1,99	0,18	7,95E-08	CG31445 (4156)	SP1029
3R	4183130..4183216	86	0,32	0,14	8,07E-08		sr (9733308)
3L	223909..223980	71	0,08	0,29	9,24E-08		CG10426 (12046812)
2R	1227850..1227889	39	0,77	0,23	1,07E-07		mspo (9331550)
2L	10339566..10339602	36	3,78	0,2	1,10E-07	SamDC	CG31715 (2018)
2L	5059638..5059693	55	0,12	0,29	1,40E-07		CG11030 (664964)
3R	3953372..3953421	49	0,82	0,16	1,40E-07		sr (9963103)
3L	20395021..20395074	53	1,03	0,09	1,46E-07	trbl (313)	CG13248 (5126)
3L	3461466..3461520	54	0,62	9,35E-03	1,51E-07		CG10426 (8809272)
chrX	8358830..8358873	43	9,00E-03	0,21	1,91E-07	Es2 (1412)	Sptr
3L	12138807..12138871	64	1,26	0,14	2,00E-07		CG10426 (131921)
3R	14069490..14069524	34	1,94	8,26E-03	2,16E-07	14-3-3epsilon	CG18598 (7311)
3L	19288396..19288436	40	2,52	0,02	2,22E-07	fal	pip (3193)
2R	12907739..12907787	48	0,08	0,13	2,52E-07	Ark	RhoGEF2 (6958)
2R	5956060..5956100	40	0,92	0,14	2,71E-07		mspo (4603339)
2R	14328572..14328619	47	0,97	0,08	2,74E-07	slim	CG30120 (5455)
chrX	8304790..8304828	38	1,4	0,15	2,82E-07	Trf2	CG32712 (22288)
3R	21155308..21155377	69	0,63	0,13	3,16E-07	CHKov1 (284)	CHKov2
chrX	2553026..2553070	44	1,17	0,07	3,26E-07		CG33080 (3049523)
2L	453519..453551	32	0,18	0,17	3,38E-07		CG11030 (5271106)

3R	26425283..26425323	40	0,11	0,07	3,51E-07	CG9717 (12262)	CG2246
3L	20369446..20369489	43	3	0,15	3,73E-07	RhoBTB	CG5498 (6015)
3R	24859556..24859607	51	1,42	0,2	3,76E-07	Ssl2 (1301)	beta4GalNAcTB
2R	13308658..13308688	30	0,08	0,33	3,99E-07	l(2)k01209	CG6550 (4479)
3L	14975991..14976057	66	2,29	0,4	4,30E-07	CG5114	mnd (2157)
3R	3362346..3362392	46	0,82	0,08	4,31E-07		sr (10554132)
chrX	16971462..16971488	26	0,39	0,33	4,86E-07	CG34326 (44894)	CG8949
2L	8685802..8685882	80	0,05	0,15	4,87E-07	tRNA:CR31888 (19:	PrBP
chrX	14969363..14969409	46	1,87	0,03	5,51E-07	Ir10a (3764122)	CG4928 (1837821)
2L	10388585..10388616	31	2,14	0,05	5,90E-07	da	CG5362 (5230)
chrX	20915601..20915655	54	0,35	0,34	6,38E-07	Mgstl (460)	CG1753 (509)
2R	10640131..10640189	58	0,01	0,29	6,50E-07	AttB (2461)	Rpn6
3R	25048952..25049007	55	2,26	0,1	6,52E-07	Ef1gamma (1117)	CG1458
chrX	15885418..15885458	40	1,36	0,05	6,85E-07	Ir10a (4680177)	CG4928 (921772)
3R	3833674..3833705	31	0,25	0,21	6,95E-07		sr (10082819)
chrX	5774421..5774457	36	1,97	0,17	7,57E-07	CG16721	Act5C (20439)
2R	13306931..13306985	54	2,69	0,03	8,00E-07	Prosalpha5 (5657)	cnk
chr4	214067..214097	30	2,5	0,07	8,00E-07	CG2219 (14087)	CG2316
3R	5616759..5616813	54	1,29	0,07	8,03E-07		sr (8299711)
2L	2216160..2216204	44	0,77	0,1	8,29E-07		CG11030 (3508453)
3R	12473367..12473421	54	2,55	0,24	8,50E-07		sr (1443103)
3R	27045181..27045218	37	2,74	0,38	8,54E-07	CG15561 (3553)	CG1746
2L	4403368..4403423	55	0,25	0,24	8,79E-07		CG11030 (1321234)
2R	5173911..5173950	39	0,05	0,12	9,38E-07		mspo (5385489)
2R	20495363..20495402	39	1,31	0,21	9,78E-07	CG42361	CG4741 (2954)
3L	8594476..8594522	46	1,2	0,08	9,78E-07		CG10426 (3676270)
3R	27619414..27619456	42	1,86	0,33	1,03E-06	RpL6 (197447)	
2L	22125919..22125962	43	0,06	0,03	1,07E-06	CG31619 (396868)	
chrX	5583338..5583385	47	2,28	0,15	1,08E-06		CG33080 (19208)
3R	19821487..19821528	41	1,39E-03	0,34	1,14E-06	CG33108	LSm3 (455)
3L	19785202..19785262	60	2,1	0,06	1,15E-06	trpml (73752)	CG42637
2L	1150884..1150925	41	2,14	0,27	1,17E-06		CG11030 (4573732)
2R	11198465..11198499	34	0,21	0,15	1,18E-06	CG8102 (12631)	CG8152
3L	5756786..5756847	61	1,18	0	1,22E-06		CG10426 (6513945)
3R	7835976..7836010	34	0,65	0,15	1,24E-06		sr (6080514)
3R	26213233..26213286	53	4,39E-03	0,27	1,25E-06	CG34300 (80871)	Fer1HCH
3L	8714769..8714826	57	4,11E-03	0,08	1,27E-06		CG10426 (3555966)
2R	717442..717493	51	0,08	0,06	1,30E-06		mspo (9841946)
2L	3813230..3813270	40	0,19	0,11	1,35E-06		CG11030 (1911387)
3R	27230304..27230334	30	3,81	0,08	1,37E-06	Gcn2 (2569)	CG11337
3R	7388411..7388463	52	2,11	0,11	1,40E-06		sr (6528061)
chrX	8009706..8009749	43	0,18	0,3	1,42E-06	CG2258 (13619)	Gclc
2R	19441602..19441644	42	0,37	0,1	1,45E-06	levy (5097)	pita
3L	10891640..10891689	49	1,66	0,09	1,46E-06		CG10426 (1379103)
2L	20175462..20175580	118	9,25E-03	0,1	1,53E-06	CG10651 (40561)	CG40463 (50294)
3L	19056846..19056870	24	1,48	0,15	1,55E-06	CG6812	Mkp3 (3961)
2R	14502671..14502720	49	1,35	0,29	1,56E-06	CG5323	CG5327 (772)
3R	19598879..19598923	44	2,98	0,02	1,85E-06	tst (350)	CG10208
2L	10270163..10270200	37	1,59	0,19	1,90E-06	CG31873	trk (1242)
3L	8989423..8989473	50	0,91	0,22	1,93E-06		CG10426 (3281319)
3L	20759499..20759556	57	0,08	0,2	1,94E-06	CG33912 (10714)	CG4074 (1950)
3R	20123993..20124048	55	0,27	0,17	2,04E-06	crb	CG5720 (16333)
3R	11055479..11055538	59	0,11	0,35	2,07E-06		sr (2860986)
2L	21685755..21685824	69	0,11	0,05	2,08E-06	CG31619	step (55429)
2L	10517530..10517575	45	0,07	0,11	2,13E-06	Lrr47	Lip4 (11841)
2L	17473024..17473067	43	2,53	0,25	2,13E-06	CG18563 (14999)	BicD

3R	8452523..8452564	41	1,66	0,16	2,13E-06		sr (5463960)
2L	6048176..6048215	39	0,93	0,08	2,22E-06	ade2 (2206)	slmo
3R	3951800..3951854	54	1,53	0,04	2,25E-06		sr (9964670)
chrX	7952401..7952432	31	0,45	0,31	2,46E-06	CG2233 (20868)	fs(1)h
2R	10414307..10414353	46	1,78	0,31	2,51E-06		mspo (145086)
chrX	2579314..2579371	57	0,57	0,16	2,51E-06		CG33080 (3023222)
3L	1653114..1653145	31	0,08	0,06	2,51E-06		CG10426 (10617647)
2L	13220102..13220141	39	4,75	0,18	2,52E-06	CG6488 (1720983)	beat-IIIc (3969668)
3R	25761363..25761411	48	1,24	0,04	2,66E-06	CG42558 (1054)	eIF2B-alpha
chrX	8355825..8355908	83	3,13E-10	0,03	2,70E-06	Es2	Sptr (1429)
3R	20046164..20046203	39	0,11	0,06	2,77E-06	CG6204 (23555)	twin
3L	16221635..16221681	46	1,81	0,06	2,79E-06	tRNA:CR32153 (64	CG5018
2L	8957429..8957512	83	0,1	0,04	2,86E-06	C1GalTA (6229)	CG31886
2L	16288374..16288440	66	1,32	0,2	2,88E-06	CG6488 (4789255)	beat-IIIc (901369)
3L	2258047..2258078	31	0,29	0,1	2,88E-06		CG10426 (10012714)
2L	10408538..10408579	41	0,9	0,2	2,92E-06	lp259	CG5355 (1957)
2R	18885250..18885289	39	2,31	0,1	2,92E-06	CG9897 (11399)	CG9896
3R	20397237..20397273	36	0,95	0,09	2,97E-06	snRNA:U6:96Ac (1	CG13624
2R	12037833..12037879	46	0,03	0,19	3,00E-06	Ext2 (712)	CG10734 (7036)
2L	20864298..20864332	34	2,54	0,1	3,03E-06	CR9337 (523)	CG9338 (894)
2R	14022708..14022758	50	0,27	0,3	3,09E-06	Hsf (572)	Pcl (175)
3R	1414903..1414943	40	0,19	0,07	3,10E-06		sr (12501581)
3L	7332709..7332762	53	1,66	0,1	3,18E-06		CG10426 (4938030)
chr4	680145..680200	55	0,34	0,08	3,19E-06	CG9935 (12963)	gw
2L	8001757..8001835	78	0,18	0,11	3,28E-06	CG7224 (2014)	Wwox
3R	11789750..11789788	38	0,11	0,3	3,38E-06		sr (2126736)
2R	8232766..8232819	53	1,08	0,35	3,42E-06		mspo (2326620)
2L	21221668..21221702	34	1,8	0,1	3,48E-06	CG8677	CG31626 (10781)
3R	14049762..14049830	68	0,1	0,01	3,53E-06	CG7993	CG7168 (793)
3R	20949958..20950011	53	1,29	0,02	3,64E-06	vig2	CG42503 (1342)
2L	17448370..17448414	44	1,09	0,3	3,87E-06	CG33928 (11770)	dl
3R	559534..559599	65	1,53	0,03	3,97E-06		sr (13356925)
2R	12118037..12118083	46	0,09	0,07	4,26E-06	CG7798 (7905)	CG15706
2L	8950477..8950502	25	0,11	0,1	4,35E-06	CG9515 (7213)	C1GalTA
3R	23408059..23408118	59	2,78	0,23	4,87E-06	wdb (98)	raps (408)
2L	19472621..19472694	73	0,84	0,35	4,97E-06	tj (4863)	CG10195 (10173)
2R	10870200..10870229	29	0,79	0,01	5,08E-06	pcs	tRNA:CR30241 (1602)
2L	479423..479472	49	1,59	0,21	5,10E-06		CG11030 (5245185)
3R	9857086..9857123	37	0,07	0,05	5,14E-06		sr (4059401)
2L	2492464..2492512	48	1,21	0,04	5,20E-06		CG11030 (3232145)
3R	26713326..26713381	55	2,05	0,03	5,25E-06	CG15547 (4101)	Sap-r
chrX	12630803..12630846	43	0,4	0,15	5,29E-06	Ir10a (1425562)	CG4928 (4176384)
chrX	12553700..12553723	23	3,68	0,02	5,32E-06	Ir10a (1348459)	CG4928 (4253507)
3R	2485656..2485703	47	0,53	0,06	5,37E-06		sr (11430821)
chrX	10660251..10660310	59	0,41	0,04	5,46E-06	Tango5 (1982)	Atg8a
3L	21209646..21209670	24	0,11	0,03	5,50E-06	CG10508 (492)	CG12975
2L	10414808..10414865	57	1,04	0,14	5,53E-06	Klp31E	RfC3 (4324)
chrX	18410007..18410054	47	0,48	0,17	5,59E-06	Aats-his	CG15048 (4808)
chrX	530444..530486	42	3,01	0,12	5,68E-06		CG33080 (5072107)
2R	5323820..5323863	43	0,6	0,1	5,79E-06		mspo (5235576)
3L	21274428..21274475	47	0,16	0,33	5,85E-06	AcCoAS (1085)	ppl
chr4	887376..887417	41	0,23	0,3	6,25E-06	CG33797 (22946)	CG11148
2R	19244422..19244461	39	0,36	9,52E-03	6,37E-06	CG30410 (707)	CG3500
3R	5572858..5572889	31	0,11	0,11	6,60E-06		sr (8343635)
chrX	7951504..7951551	47	0,03	0,06	6,74E-06	CG2233 (19971)	fs(1)h
2R	17554718..17554756	38	1,39	0,26	6,76E-06	CG42365	CG42379 (289)

2L	10002182..10002223	41	0,4	0,28	6,83E-06	CG31875 (5610)	CG31755
chrX	8609051..8609093	42	2,44	0,36	6,88E-06	CG1785	l(1)G0020 (1653)
2L	6917371..6917392	21	3,27	0,05	7,17E-06	wee (3234)	xl6
3R	5595507..5595556	49	0,07	0	7,27E-06		sr (8320968)
3L	3142912..3142978	66	0,16	0,08	7,40E-06		CG10426 (9127814)
2L	6782919..6782947	28	1,53	0,11	7,47E-06	CG17378 (4463)	nrv1
2R	9727088..9727151	63	0,51	0,17	7,50E-06		mspo (832288)
2R	4558456..4558510	54	0,38	0,05	7,63E-06		mspo (6000929)
chrX	6493213..6493245	32	2,02	0,05	7,76E-06	APC7	CG3973 (852)
3R	6038270..6038316	46	2,11	0,06	7,82E-06		sr (7878208)
2L	1150296..1150329	33	1,19	0,26	7,93E-06		CG11030 (4574328)
2L	7828589..7828638	49	0,3	0,28	8,12E-06	mts	CG14537 (11842)
2R	19590137..19590185	48	0,19	0,02	8,21E-06	l(2)dtl	l(2)not (2774)
2L	21623340..21623395	55	1,76E-04	0,11	8,37E-06	CR42546 (32747)	CG2201
3R	27434641..27434665	24	1	0,23	8,39E-06	RpL6 (12674)	CycG
chrX	8383068..8383112	44	0,21	0,11	8,40E-06	Cp38 (5724)	otu
3R	13366973..13367005	32	1,14	0,01	8,45E-06		sr (549519)
2L	4851020..4851048	28	3,33	0,1	8,65E-06		CG11030 (873609)
chrX	21533009..21533051	42	0,03	0,1	8,72E-06	DIP1 (31824)	CG14621 (313538)
3R	7606735..7606774	39	1,23	0,14	8,80E-06		sr (6309750)
2L	21162884..21162913	29	1,62	0,04	8,94E-06	CG9248 (2101)	CG9247
3R	8234259..8234285	26	0,66	0,03	8,98E-06		sr (5682239)
3L	7866356..7866393	37	1,21	0,17	9,19E-06		CG10426 (4404399)
2L	4442505..4442547	42	0,03	0,33	9,73E-06		CG11030 (1282110)
chrX	2009055..2009126	71	1,15	0,27	9,94E-06		CG33080 (3593467)
2L	19426173..19426247	74	7,26E-03	0,09	1,01E-05	CG31798 (29983)	Pax
2R	6454596..6454622	26	1,76	0,09	1,01E-05		mspo (4104817)
2R	13308066..13308097	31	2,08	0,23	1,02E-05	l(2)k01209	CG6550 (5070)
3L	1777583..1777620	37	0,05	0,03	1,03E-05		CG10426 (10493172)
3R	3575571..3575639	68	0,18	0,09	1,05E-05		sr (10340885)
chrX	10657330..10657367	37	2,59	0,1	1,10E-05	Rph (5631)	Tango5
2L	8082412..8082440	28	3,86	0,05	1,12E-05	Trf (8463)	Mcr
3R	22060147..22060222	75	0,02	0,05	1,12E-05	CG12290 (1077)	Ald (20181)
3R	17704259..17704289	30	2,83	0,15	1,12E-05	CG6656 (749)	CG34148
2R	5988915..5988953	38	0,5	0,16	1,14E-05		mspo (4570486)
3R	7793432..7793468	36	3,36	0,2	1,16E-05		sr (6123056)
2R	10062767..10062808	41	2,94	0,09	1,17E-05		mspo (496631)
2L	10544972..10545117	145	8,25E-03	0,03	1,21E-05	Trim9	CG6138 (22172)
2L	478858..478906	48	0,41	0,19	1,23E-05		CG11030 (5245751)
3R	4859664..4859728	64	0,19	0,16	1,23E-05		sr (9056796)
3R	7575061..7575107	46	2,83	0,02	1,24E-05		sr (6341417)
3R	3563599..3563644	45	0,64	0,03	1,25E-05		sr (10352880)
2L	844130..844172	42	2,17	0,12	1,30E-05		CG11030 (4880485)
2L	21618196..21618261	65	0,7	0,33	1,33E-05	CR42546 (27603)	CG2201
3R	19045447..19045520	73	1,24E-04	0,13	1,34E-05	Ir (34083)	cnc
chrX	15476874..15476911	37	0,29	0,22	1,35E-05	Ir10a (4271633)	CG4928 (1330319)
3L	12074645..12074677	32	2,65	8,00E-03	1,35E-05		CG10426 (196115)
3L	2257779..2257849	70	0,05	0,3	1,40E-05		CG10426 (10012943)
chrX	517840..517865	25	0,95	0,17	1,41E-05		CG33080 (5084728)
3L	19578934..19578975	41	1,69	0,2	1,42E-05	Shal (1254)	CG9231
3R	23109647..23109677	30	2,11	0,13	1,45E-05	CG3368	bigmax (3409)
chrX	4003375..4003421	46	7,50E-03	0,23	1,51E-05		CG33080 (1599172)
2R	4542186..4542223	37	1,39	0,14	1,55E-05		mspo (6017216)
2L	13572867..13572910	43	3,13	0,13	1,55E-05	CG6488 (2073748)	beat-IIIc (3616899)
2R	13759293..13759330	37	3,01	0,03	1,58E-05	thr (5896)	Mapmodulin
2L	10356345..10356391	46	2,2	0,25	1,58E-05	CG34367 (2714)	CG5367

chrX	21948143..21948192	49	1,78	0,12	1,59E-05	CG14619 (77816)	
3L	15809133..15809174	41	1,29	0,35	1,60E-05	CG13445 (6150)	CG6151
3L	16227656..16227719	63	0,13	0,13	1,63E-05	CG32155	CG32154 (3138)
2R	12614236..12614288	52	2,33	0,11	1,63E-05	CG34458 (53906)	CG30463
3R	3361819..3361863	44	1,41	0,26	1,68E-05		sr (10554661)
2R	5009968..5009992	24	1,56	0,15	1,69E-05		mspo (5549447)
3L	8722735..8722761	26	0,15	0,08	1,76E-05		CG10426 (3548031)
3R	2904821..2904862	41	0,72	0,15	1,76E-05		sr (11011662)
3L	22064819..22064859	40	0,4	0,15	1,80E-05	CG14561 (4752)	CG7139
3L	6948803..6948840	37	1,06	0,24	1,81E-05		CG10426 (5321952)
2L	9706259..9706319	60	0,25	0,15	1,81E-05	CG31710 (315)	CG4364 (2600)
3L	18674782..18674844	62	0,81	0,03	1,81E-05	CG34254 (903)	Sgf11
3R	5567177..5567209	32	0,4	0,15	1,85E-05		sr (8349315)
3L	2273909..2273968	59	2,16	0,04	1,86E-05		CG10426 (9996824)
2L	10264192..10264236	44	0,61	0,17	1,86E-05	CG18619	Mob3 (1102)
3L	1303881..1303925	44	0,81	0,05	1,88E-05		CG10426 (10966867)
chrX	19074512..19074534	22	2,38	0,13	1,95E-05	CG7453	CG33253 (2922)
2R	13565849..13565898	49	0,45	0,19	1,95E-05	CG6459 (5701)	Sema-1b
2L	10363538..10363584	46	2,35	0,32	1,97E-05	pim	CG13139 (1533)
3R	137665..137697	32	0,11	0,1	1,99E-05		sr (13778827)
3L	4825985..4826035	50	0,13	0,2	2,01E-05		CG10426 (7444757)
3L	5127786..5127822	36	1,64	0,05	2,06E-05		CG10426 (7142970)
2R	6698284..6698314	30	2,25	0,18	2,14E-05		mspo (3861125)
3R	16658919..16658961	42	0,64	0,15	2,20E-05	Synd	CG15695 (8902)
2L	8083528..8083571	43	2,35	0,14	2,22E-05	Bsg	CG8683 (33393)
3R	7235076..7235118	42	2,89	0,27	2,23E-05		sr (6681406)
2R	9709574..9709622	48	0,3	0,16	2,24E-05		mspo (849817)
chrX	8376338..8376403	65	0,72	0,1	2,25E-05	Sptr (17308)	CG33223
3L	20519772..20519813	41	4,04	0,3	2,26E-05	CG4858	Rcd2 (1254)
chrX	3675350..3675405	55	0,97	0,3	2,26E-05		CG33080 (1927188)
2R	17521610..17521661	51	0,56	0,2	2,27E-05	CG15674	CG10321 (511)
2R	9120292..9120357	65	0,68	0,12	2,30E-05		mspo (1439082)
2L	17870968..17871112	144	0,04	0,15	2,36E-05	CadN2 (54007)	btv (91622)
2L	13370854..13370893	39	2,22	0,26	2,38E-05	CG6488 (1871735)	beat-IIIc (3818916)
2L	8400615..8400658	43	2,05	0,15	2,43E-05	CG31898	fy (787)
chrX	7202375..7202414	39	2,23	0,03	2,47E-05	brk	Atg5 (6233)
2L	2886600..2886626	26	2,12	0,08	2,54E-05		CG11030 (2838031)
chrX	6557..6594	37	3,90E-20	0,05	2,62E-05		CG33080 (5595999)
chrX	9529888..9529934	46	0,27	0,06	2,67E-05	CG3099 (14188)	l(1)G0232
3R	20677389..20677426	37	0,95	0,21	2,69E-05	nct	HdacX (3354)
chrX	9449423..9449445	22	0,21	0,11	2,70E-05	RpS28b (169)	CG15317 (194)
2R	9122308..9122389	81	0,32	0,08	2,70E-05		mspo (1437050)
2L	18688442..18688470	28	1,49	0,11	2,75E-05	tos (6287)	msl-1
3R	8411394..8411446	52	1,37	0,03	2,77E-05		sr (5505078)
2R	6317989..6318034	45	2,18	0,15	2,81E-05		mspo (4241405)
3R	7584095..7584154	59	1,33	0,28	2,85E-05		sr (6332370)
3L	7352926..7352949	23	0,12	0,02	2,86E-05		CG10426 (4917843)
2R	8869069..8869107	38	2,39	0,07	2,88E-05		mspo (1690332)
2L	6507293..6507347	54	0,22	0,15	2,88E-05	CG31637	eya (20101)
3R	20032626..20032764	138	0,18	0,04	2,89E-05	CG6204 (10017)	twin
2R	8759791..8759841	50	0,15	0,06	2,90E-05		mspo (1799598)
3R	13718812..13718850	38	1,35	0,28	2,92E-05		sr (197674)
chrX	8801651..8801690	39	0,48	0,01	2,92E-05	e(r) (1374)	CG15352 (57)
2R	12950473..12950502	29	1,91	0,08	2,95E-05	CG6967	CG30460 (2973)
chrX	21221161..21221201	40	0,73	0,03	2,98E-05	pen (295)	sol (155)
chrX	19249190..19249234	44	1,82	0,14	3,01E-05	kek5	CG32533 (118943)

3L	5773521..5773543	22	1,45	0,13	3,02E-05		CG10426 (6497249)
chrX	2608962..2609005	43	0,35	0,17	3,11E-05		CG33080 (2993588)
chrX	19625429..19625478	49	0,18	0	3,12E-05	Bap	CG14234 (4128)
2L	10356182..10356218	36	0,57	0,08	3,15E-05	CG34367 (2551)	CG5367
3L	21001815..21001860	45	2,72	0,22	3,15E-05	CG12984 (15978)	skd
2R	9325981..9326009	28	0,52	0,16	3,21E-05		mspo (1233430)
3L	3250242..3250276	34	0,45	0,33	3,23E-05		CG10426 (9020516)
3R	2260119..2260173	54	0,5	0,25	3,25E-05		sr (11656351)
3L	22483842..22483903	61	1,01	0,07	3,29E-05	CG14561 (423775)	
chrX	11749457..11749485	28	3,5	0,17	3,42E-05	Ir10a (544216)	CG4928 (5057745)
3L	1006898..1006951	53	0,61	0,08	3,55E-05		CG10426 (11263841)
3L	17945126..17945186	60	0,11	0,35	3,56E-05	CG5290 (39469)	Eip75B
3R	27269983..27270031	48	0,99	0,25	3,59E-05	Gprk2	lox (14274)
3R	14025158..14025214	56	0,85	0,12	3,61E-05	CG14313 (7469)	Ssdp
2R	8318439..8318489	50	0,33	0,28	3,62E-05		mspo (2240950)
3R	5959748..5959777	29	0,54	0,04	3,64E-05		sr (7956747)
2L	12045489..12045531	42	2,36	0,14	3,76E-05	CG6488 (546370)	beat-IIIc (5144278)
3L	17637435..17637462	27	2,05	0,19	3,82E-05	CG7497 (6395)	Pep
3L	8118102..8118139	37	3,02	0,2	3,85E-05		CG10426 (4152653)
3R	19519120..19519173	53	2,41	0,04	3,96E-05	SPE (5247)	CG10254
3R	15230331..15230371	40	0,64	0,13	3,97E-05	CG5316	CG11626 (4525)
3R	19520311..19520353	42	2,73	0,09	3,99E-05	SPE (6438)	CG10254
3R	14069252..14069306	54	1,19E-04	0,05	4,06E-05	14-3-3epsilon	CG18598 (7529)
3R	7836636..7836669	33	2,71	0,17	4,13E-05		sr (6079855)
chrX	7786623..7786671	48	1,82	0,2	4,13E-05	Nek2 (1018)	CG18624 (414)
2L	21611079..21611157	78	0,01	0	4,20E-05	CR42546 (20486)	CG2201 (3605)
2L	19886..19950	64	4,99E-04	0,2	4,26E-05		CG11030 (5704707)
3L	11089699..11089745	46	1,45	0,35	4,35E-05		CG10426 (1181047)
2L	8998199..8998231	32	1,76	0,39	4,35E-05	CG18661	Try29F (305)
3R	20655512..20655551	39	0,82	0,03	4,41E-05	RabX4 (1821)	CG31357
3R	4169485..4169527	42	1,86	0,34	4,46E-05		sr (9746997)
3R	9208055..9208102	47	1	0,03	4,46E-05		sr (4708422)
chrX	2052249..2052286	37	2,32	0,33	4,58E-05		CG33080 (3550307)
2R	485863..485935	72	1,31	0,08	4,63E-05		mspo (10073504)
2L	346951..347047	96	9,72E-05	0,03	4,64E-05		CG11030 (5377610)
2R	20972945..20972981	36	0,97	0,18	4,68E-05	CG12851 (130941)	
3R	23745001..23745045	44	1,21	0,14	4,70E-05	mRpS22 (1031)	CG33213
2R	12950727..12950765	38	0,81	0,12	4,76E-05	CG6967	CG30460 (2710)
chrX	11463492..11463517	25	2,59	0,07	4,82E-05	Ir10a (258251)	CG4928 (5343713)
3L	8434655..8434687	32	0,28	0,12	4,82E-05		CG10426 (3836105)
3R	25615628..25615675	47	0,04	0,25	4,87E-05	fig (9454)	CG12068 (4682)
3L	7236817..7236861	44	0,33	0,15	4,89E-05		CG10426 (5033931)
3L	11077086..11077128	42	2,13	0,1	4,89E-05		CG10426 (1193664)
3R	7505250..7505293	43	0,16	0,07	4,93E-05		sr (6411231)
chrX	15707240..15707273	33	0,95	0,14	4,97E-05	Ir10a (4501999)	CG4928 (1099957)
3R	20891263..20891321	58	0,63	0,26	5,00E-05	CG34349 (1272)	veli
chrX	8311915..8311953	38	1,95	0,07	5,00E-05	Trf2	CG32712 (15163)
3R	18550061..18550091	30	1,36	0,03	5,04E-05	wge (3784)	Irp-1A
2R	5054183..5054238	55	1,36	0,1	5,06E-05		mspo (5505201)
2R	15017399..15017441	42	0,1	0,22	5,13E-05	botv	CG15118 (3780)
3L	20835214..20835251	37	0,05	0,12	5,15E-05	CG11456	CG32432 (2173)
2L	18571745..18571783	38	0,02	0,13	5,17E-05	Pde11 (97)	CG15160 (19113)
chrX	15700599..15700635	36	1,96	0,3	5,21E-05	Ir10a (4495358)	CG4928 (1106595)
3R	7446042..7446070	28	1,43	0,05	5,29E-05		sr (6470454)
3R	267830..267878	48	0,85	0,09	5,35E-05		sr (13648646)
2L	21663701..21663733	32	0,22	0,03	5,36E-05	cul-2 (483)	CG2225 (58)

3R	25761792..25761841	49	1,21	0,22	5,49E-05	CG15523	Takr99D (14676)
3L	16567629..16567671	42	3,6	0,18	5,49E-05	Mipp1 (1199)	mbf1 (124)
chrX	8377136..8377170	34	2,5	0,07	5,49E-05	Sptr (18106)	CG33223
2L	11282520..11282581	61	0,31	0,07	5,50E-05	mre11	Osi21 (2004)
2L	19116556..19116598	42	0,18	0,15	5,52E-05	CG10561	Ddc
2R	8481382..8481409	27	0,1	0,11	5,56E-05		mspo (2078030)
3R	24957527..24957572	45	2,83	0,34	5,63E-05	Vha100-1	CG14512 (6119)
2R	20636803..20636863	60	0,83	0,02	5,67E-05	CG30421	spz6 (8039)
3L	7353774..7353826	52	0,66	0,09	5,69E-05		CG10426 (4916966)
3R	20719210..20719280	70	0,85	0,05	5,69E-05	CG31381 (14703)	CG31121
3R	10522188..10522220	32	0,61	0,05	5,70E-05		sr (3394304)
3R	14127224..14127252	28	2,71	0,29	5,77E-05	Dlc90F (1460)	CG18600
2L	19034862..19034886	24	1,8	0,11	5,78E-05	mib2	Catsup (6827)
2L	6599495..6599556	61	0,3	0,14	5,83E-05	CG13766 (32908)	CG11319
2R	12469532..12469567	35	2,17	0,07	5,85E-05	Cdk4	RpLP2 (4031)
chrX	160924..160965	41	0,56	0,17	5,85E-05		CG33080 (5441628)
3R	18983443..18983480	37	0,3	0,33	5,93E-05	hh (15562)	unk
3L	9066526..9066570	44	0,42	0,29	5,99E-05		CG10426 (3204222)
2L	2152427..2152468	41	3,02	0,18	6,00E-05		CG11030 (3572189)
chrX	14128622..14128675	53	6,48E-03	0,18	6,04E-05	Ir10a (2923381)	CG4928 (2678555)
3L	14220226..14220260	34	5,15E-03	0,25	6,12E-05	CG7768 (7836)	CG7924 (6308)
3L	16859787..16859833	46	2,12	0,29	6,12E-05	Lmpt	CG32170 (10585)
2L	19548395..19548440	45	0,89	0,03	6,18E-05	CG10337 (1481)	TepIV (1352)
chrX	20108756..20108791	35	0,45	0,28	6,23E-05	sw (1300)	obst-A (2142)
chrX	8805..8830	25	1,35	0,1	6,24E-05		CG33080 (5593763)
chrX	21392386..21392419	33	2,47	0,01	6,27E-05	Cyp6t1 (2190)	CG14476 (79207)
chrX	18410381..18410411	30	3,22	0,07	6,29E-05	Aats-his	CG15048 (4451)
2R	7088228..7088257	29	2,34	0,04	6,33E-05		mspo (3471182)
3L	21627356..21627392	36	1,35	0,23	6,36E-05	CG11306 (5940)	MED1
chrX	10636481..10636505	24	1,03	0,1	6,46E-05	CG12637 (69080)	CG32676
chr4	715521..715552	31	1,05	0,22	6,48E-05	CG11360 (8725)	myoglianin
2R	20288653..20288687	34	0,97	0,28	6,49E-05	mAcR-60C (11418)	slik
2R	6693884..6693915	31	0,97	0,22	6,53E-05		mspo (3865524)
2L	5983010..5983036	26	1,41	0,18	6,56E-05	eIF-4a	ifc (3287)
3R	19750907..19750938	31	2,27	0,07	6,60E-05	CG5857	CG13601 (1155)
3L	3018400..3018496	96	5,15E-03	0,2	6,71E-05		CG10426 (9252296)
2R	16717735..16717808	73	0,18	0,12	6,74E-05	CG15225 (36544)	insc
chrX	13644100..13644142	42	0,33	0,07	6,82E-05	Ir10a (2438859)	CG4928 (3163088)
3R	5174873..5174904	31	0,1	0,2	6,88E-05		sr (8741620)
3L	16578054..16578095	41	1,55	0,21	6,89E-05	CG4169	Syx8 (1231)
chrX	16477006..16477052	46	2,11	0,08	6,96E-05	Ir10a (5271765)	CG4928 (330178)
chrX	21860348..21860404	56	1,89	0,08	6,96E-05	CG14619	CG14613 (11689)
2R	20668595..20668628	33	0,07	0,04	7,01E-05	Eap	CG16912 (615)
3L	18385674..18385739	65	5,15E-03	0,04	7,02E-05	grim (88160)	rpr (4913)
2L	7406918..7406972	54	0,65	0,14	7,04E-05	CG5177	CG5181 (1560)
chrX	9512377..9512423	46	0,84	0,11	7,06E-05	CG3003 (5739)	CG3099 (1362)
2L	1722958..1723006	48	1,5	0,36	7,06E-05		CG11030 (4001651)
chrX	11463965..11463996	31	2,6	0,03	7,08E-05	Ir10a (258724)	CG4928 (5343234)
3L	16105705..16105757	52	0,09	0,14	7,12E-05	Zn72D (217)	Taf4 (565)
3R	11789249..11789298	49	2,02	0,09	7,12E-05		sr (2127226)
2R	793322..793348	26	0,97	0,16	7,15E-05		mspo (9766091)
2R	3819675..3819697	22	0,75	0,11	7,16E-05		mspo (6739742)
3L	9413368..9413390	22	2,81	0	7,16E-05		CG10426 (2857402)
chrX	65301..65367	66	0,04	0,14	7,17E-05		CG33080 (5537226)
chrX	9058535..9058572	37	2,59	0,21	7,19E-05	mei-P26	CG12115 (24783)
chrX	6427037..6427062	25	0,23	0,04	7,20E-05	Spat (2278)	RpL7A

chrX	11572804..11572850	46	3,98	0,23	7,22E-05	Ir10a (367563)	CG4928 (5234380)
3L	9360229..9360261	32	2,02	0,07	7,22E-05		CG10426 (2910531)
3L	6246022..6246048	26	1,18	0,07	7,28E-05		CG10426 (6024744)
3L	1652329..1652364	35	1,05	0,17	7,38E-05		CG10426 (10618428)
3L	12806243..12806279	36	0,15	0,11	7,39E-05	CG32110 (3047)	CG10754
2L	6676652..6676704	52	2,11	0,16	7,39E-05	cort (17)	CG11329 (24)
chr4	464326..464370	44	1,64	0,24	7,51E-05	CG2052 (50310)	lgs
2L	10291821..10291854	33	0,77	0,03	7,59E-05	CG34043 (10975)	CG5604
3R	16934283..16934320	37	2	0,31	7,66E-05	rtet	Rab11 (2808)
2R	12573381..12573417	36	1,76	0,13	7,75E-05	CG34458 (13051)	CG30463
2R	2019490..2019547	57	1,74	0,07	7,75E-05		mspo (8539892)
3L	4134129..4134183	54	2,49	0,1	7,80E-05		CG10426 (8136609)
2L	20508..20535	27	2,48	0,11	7,85E-05		CG11030 (5704122)
2L	6788012..6788072	60	0,06	0,38	7,87E-05	nrv1 (1156)	nrv2 (1688)
3L	16568356..16568382	26	2,59	0,09	7,97E-05	mbf1	nxf2 (1576)
2R	19932565..19932596	31	3,25	0,15	7,99E-05	CG3173 (584)	tsr
3L	4287180..4287219	39	0,47	0,29	8,04E-05		CG10426 (7983573)
2R	12905646..12905717	71	1,28	0,38	8,12E-05	CG15609 (2255)	CG8963
3R	6687955..6687976	21	1,13	0,3	8,23E-05		sr (7228548)
chrX	21505358..21505396	38	2,5	0,02	8,30E-05	DIP1 (4173)	CG14621 (341193)
3L	17661391..17661422	31	1,96	0,22	8,32E-05	TORC (3672)	snmRNA:641 (265)
3R	3943308..3943369	61	3,52	0,07	8,33E-05		sr (9973155)
3L	14750245..14750277	32	0,9	0,11	8,41E-05	CG13471 (23628)	Trl
3L	15557108..15557139	31	0,49	0,04	8,43E-05	CG7427	CG6498 (2008)
3L	6242905..6242959	54	2,02	0,06	8,47E-05		CG10426 (6027833)
3R	1810796..1810854	58	0,82	0,1	8,47E-05		sr (12105670)
3L	8433129..8433145	16	0,8	0,03	8,49E-05		CG10426 (3837647)
chrX	5794467..5794522	55	0,69	0,06	8,52E-05	CG16721 (17034)	Act5C (374)
3L	15152318..15152364	46	1,55	0,12	8,52E-05	CG6876	CG7011 (1604)
2R	5912815..5912859	44	0,54	0,26	8,57E-05		mspo (4646580)
3L	18084300..18084372	72	1,44	0,29	8,60E-05	CG34253 (3884)	CG13698
2L	18705452..18705476	24	9,31	0,05	8,60E-05	CG10343 (995)	CG10373
chrX	19389162..19389198	36	0,41	0,17	8,65E-05	CG14199 (151)	Pfrx (339)
2L	14046740..14046795	55	0,18	0,08	8,68E-05	CG6488 (2547621)	beat-IIIc (3143014)
3L	3223840..3223859	19	0,5	0,08	8,72E-05		CG10426 (9046933)
chrX	537960..538020	60	0,87	0,15	8,78E-05		CG33080 (5064573)
3L	5182460..5182514	54	1,05	0,15	8,78E-05		CG10426 (7088278)
3R	7035788..7035824	36	0,17	0,26	8,78E-05		sr (6880700)
2L	12701679..12701722	43	1,12	0,38	8,80E-05	CG6488 (1202560)	beat-IIIc (4488087)
2R	7075079..7075110	31	0,41	0,11	8,88E-05		mspo (3484329)
3L	4287310..4287351	41	0,9	0,08	8,90E-05		CG10426 (7983441)
chr4	153715..153760	45	6,65	0,16	8,94E-05	CG32000	CG32006 (17632)
chrX	12562078..12562124	46	2,55	0,02	9,02E-05	Ir10a (1356837)	CG4928 (4245106)
3R	11117678..11117719	41	1,42	0,04	9,08E-05		sr (2798805)
3L	17954058..17954084	26	1,55	0	9,11E-05	CG5290 (48401)	Eip75B
3R	12866438..12866459	21	2,85	0,38	9,19E-05		sr (1050065)
2L	12251604..12251664	60	0,01	0,05	9,21E-05	CG6488 (752485)	beat-IIIc (4938145)
3L	213086..213113	27	1,83	0,05	9,34E-05		CG10426 (12057679)
chrX	1264580..1264606	26	1,5	0,08	9,35E-05		CG33080 (4337987)
2L	7810532..7810571	39	0,77	0,16	9,36E-05	r2d2 (8524)	cdc14
3R	4065198..4065223	25	2,55	0,15	9,45E-05		sr (9851301)
chrX	18174750..18174791	41	1,15	0,05	9,49E-05	upd3	os (24585)
3R	152461..152509	48	1,73	0,05	9,49E-05		sr (13764015)
2R	6773231..6773260	29	1,8	0,2	9,50E-05		mspo (3786179)
2L	5009200..5009220	20	1,49	0,24	9,52E-05		CG11030 (715437)
3L	347891..347920	29	0,78	7,19E-03	9,64E-05		CG10426 (11922872)

3R	7056084..7056107	23	0,5	0,02	9,65E-05		sr (6860417)
2R	19866912..19866945	33	2,38	0,13	9,75E-05	Sox14	Phm (5804)
2R	20846854..20846891	37	0,01	0,09	9,80E-05	CG12851 (4850)	CG2765 (469)
3L	11188900..11188969	69	2,15	0,12	9,85E-05		CG10426 (1081823)
2R	12705405..12705449	44	1,76	0,12	9,86E-05	CG5550 (319)	CG34459 (7587)
chrX	15396750..15396793	43	0,21	0,3	9,87E-05	Ir10a (4191509)	CG4928 (1410437)
3L	15569517..15569568	51	1,42	0,26	9,88E-05	CG6498 (664)	CG16979 (97)
